# The role of *SOD2* and *NOS2* genes in the molecular aspect of bladder cancer pathophysiology

**DOI:** 10.1038/s41598-023-41752-8

**Published:** 2023-09-02

**Authors:** Radosław Grębowski, Joanna Saluk, Michał Bijak, Janusz Szemraj, Paulina Wigner-Jeziorska

**Affiliations:** 1 Department of Medical Biochemistry, Medical University of Lodz, Lodz, Poland Mazowiecka 6/8, 90-001; 2 Department of Urology, Provincial Integrated Hospital in Plock, Plock, Poland Medyczna 19, 09-400; 3 Department of General Biochemistry, Faculty of Biology and Environmental Protection, University of Lodz, Lodz, Poland Pomorska 141/143, 90-236; 4 Biohazard Prevention Centre, Faculty of Biology and Environmental Protection, University of Lodz, Lodz, Poland Pomorska 141/143, 90-236

**Keywords:** Biochemistry, Molecular biology, Urology

## Abstract

Bladder cancer (BC) is a severe health problem of the genitourinary system and is characterised by a high risk of recurrence. According to the recent GLOBOCAN report, bladder cancer accounts for 3% of diagnosed cancers in the world, taking 10th place on the list of the most common cancers. Despite numerous studies, the full mechanism of BC development remains unknown. Nevertheless, precious results suggest a crucial role of oxidative stress in the development of BC. Therefore, this study explores whether the c. 47 C > T (rs4880)—*SOD2*, (c. 1823 C > T (rs2297518) and g.-1026 C > A (rs2779249)—*NOS2(iNOS)* polymorphisms are associated with BC occurrence and whether the bladder carcinogenesis induces changes in *SOD2* and *NOS2* expression and methylation status in peripheral blood mononuclear cells (PBMCs). In this aim, the TaqMan SNP genotyping assay, TaqMan Gene Expression Assay, and methylation‐sensitive high‐resolution melting techniques were used to genotype profiling and evaluate the expression of the genes and the methylation status of their promoters, respectively. Our findings confirm that heterozygote of the g.-1026 C > A SNP was associated with a decreased risk of BC. Moreover, we detected that BC development influenced the expression level and methylation status of the promoter region of investigated genes in PBMCs. Concluding, our results confirmed that oxidative stress, especially *NOS2* polymorphisms and changes in the expression and methylation of the promoters of *SOD2* and *NOS2* are involved in the cancer transformation initiation of the cell urinary bladder.

## Introduction

The bladder is a hollow organ located in the lower abdomen that is mainly responsible for storing urine received from the kidneys (via the ureter) until voiding. The bladder and urinary tract are lined with specialised transitional epithelial cells, known as urothelial cells that allow the urine produced to be collected by flattening under pressure. Under the epithelial layer, there are smooth muscles, which, on the one hand, enable the storage of a larger volume of urine, and on the other hand, as a result of contraction (under the control of the will or reflex), allow urine to be excreted through the urethra^1^. Therefore, the urothelial cells that line the bladder and urinary tract are constantly in contact with urine and thus exposed to environmental factors that are filtered into the urine by the kidneys^2^. Not surprisingly, 90% of bladder cancers, especially in developed countries, originate from urothelial cells^3^. Interestingly, according to the recent GLOBOCAN report, bladder cancer accounts for 3% of diagnosed cancers in the world, taking 10th place on the list of the most common cancers. Moreover, its incidence is steadily increasing worldwide, especially in developed countries^4,5^. It was estimated that in 2020, BC claimed nearly 212,536 deaths, which is 2.2% of all cancer deaths^5^.

Most cases of BC are caused by exposure to environmental and occupational chemicals, of which tobacco smoke is by far the largest^4^. Risk factors for the development of bladder cancer also include increasing age^6^, exposure to chemical agents (e.g. aromatic amines, arsenic)^7–9^, male gender^4^, obesity^10^, and low daily fluid intake (< 0.4 L/day)^11,12^. In addition to environmental factors, genetic factors are also involved in the mechanism of BC development. Previous studies confirmed that single nucleotide polymorphisms (SNPs) and mutations localised in *NAT2*, *GSTM1*, *MYC*, *TP63*, *PSCA*, *CLPTM1L-TERT*, *TACC3-FGFR3*, *APOBEC3A-CBX6*, *CCNE1*, and *UGT1A* may modulate the risk of developing bladder cancer^13^. However, despite the knowledge of numerous BC risk factors, the molecular mechanism of BC development still remains unclear. Previous studies suggest the role of the overproduction of reactive oxygen (ROS) and nitrogen species (RNS) in the process of carcinogenesis of the urinary bladder^14^. Patients with BC were characterised by lower expression of antioxidant enzymes, including superoxide dismutase 2 (SOD2) as compared to controls^15,16^. On the other hand, BC patients showed increased levels of MDA (malondialdehyde) in the serum^17^ and 8-iso-PGF2α (8-iso prostaglandin F 2α) in the urine^18^, known markers of oxidative stress as well as nitric oxide (NO, a product of nitric oxide synthetase activity) in the bladder cancer tissue, urine and serum^19,20^.

Increased production of ROS in course of BC development may be a consequence of the failure of antioxidant defence and mitochondria dysfunction^21^. Under physiological conditions, antioxidant enzymes, including SOD2, play an essential role in the first line of defence against free radicals^22^. In the process of BC carcinogenesis, as a result of increased energy activity of mitochondria, overproduction of ROS by mitochondria is observed, accompanied by disorders of enzymatic oxidative defence, including SOD2^23^. Interestingly, the altered activity of enzymes and the reduced ability to neutralise oxygen free radicals may be the result of the appearance of genetic polymorphisms in the genes encoding antioxidant defence enzymes^22^. So far, the impact of SOD2 gene polymorphisms on ROS production disorders has been well characterised. The SNP located in the *SOD2* gene (rs4880) consists of nucleotide substitutions (T, thymine → C, cytosine) and subsequent substitutions of the amino acids alanine (Ala) with valine (Val) (Ala16Val), which consequently leads to a decrease in transport efficiency in mitochondria in carriers of the Val allele by 30–40% and a reduction in the superoxide anion neutralization potential. Consequently, the appearance of this SNP is associated with reduced ROS degradation^24^.

In turn, long-term accumulation of ROS leads to damage to macromolecules, including DNA, as well as results affect the regulation of signalling pathways involved in cell proliferation, growth, survival and apoptosis. ROS overproduction also contributes to the maintenance of an inflammatory microenvironment conducive to the process of carcinogenesis^25^. ROS overproduction may activate NF-κB (nuclear factor kappa-light-chain-enhancer of activated B cells), leading to the induction of pro-inflammatory cytokines and NOS2 (nitric oxide synthase 2, so-called iNOS, inducible nitric oxide synthase) expression, which in turn exacerbate inflammation and overproduction of further ROS and RNS in a vicious circle^21^. The mentioned overproduction of ROS and RNS as well as prolonged inflammation may lead to the neoplastic transformation of cells by oxidative DNA damage, including DNA strand breaks, DNA-DNA or DNA–protein cross-linking, or stimulation of ROS/MAPK and ROS/Keap1-Nrf2-ARE as well ROS/PI3K/Akt signalling pathways associated with the promotion or inhibition of BC cell proliferation, migration, and invasion^26–30^. Interestingly, the study as part of the Cancer Genome Atlas (TCGA) project (TCGA) confirmed the crucial role of mutations localised in genes encoding Pi3K/Akt pathway factors, cell cycle regulators and apoptosis proteins in the development and progression of the BC. The course of all these processes is modulated by a number of factors, including the level of ROS and RNS. As a consequence, all abnormalities leading to disturbances in the functioning of these pathways, including disorders in the functioning of antioxidant enzymes as well as ROS and RNS overproduction, may affect the mechanism of BC development^31^.

Moreover, previous studies also show significant *NOS2* overexpression in cells of various cancers, including BC cells. In turn, high levels of NO, a product of NOS2 activity, can stimulate cell growth, dilate tumour vessels to maintain blood supply to the tumour, and thus is crucial for tumour angiogenesis. Further studies have shown that NO controls angiogenesis by modulating the activity of angiogenic factors released by tumour cells, such as vascular endothelial growth factor, which requires a functioning NO/cyclic guanosine monophosphate pathway in the endothelial compartment to promote neovascular growth and plays a key role in the angiogenic cascade, and thus is crucial for the development and progression of cancers, including BC^32,33^. As in the case of *SOD2*, previous studies have shown that polymorphisms located in *NOS2* can affect the activity of the protein. c.1823 C > T (p.Ser608Leu) (rs2297518) SNP may impact on NOS2 activity. The substitution from serine to leucine may contribute to increased NOS2 activity. In turn, g.-1026 C > A (rs2779249) polymorphism may modulate the NOS2 expression. A carriers were characterised by elevated *NOS2* promoter transcriptional activity. Thus, as a consequence of the appearance of these SNPs in the NOS2 gene, patients are characterised by an increased concentration of NO, and thus show an increased risk of carcinogenesis^34^.

Considering the reports presented above, our presented study aimed to determine the link between SNPs of *SOD2* and *NOS2* genes potentially associated with altered susceptibility to oxidative/nitrative stresses and BC prevalence. In addition, we assessed the impact of BC development on the level of *SOD2* and *NOS2* expression and methylation status of the promoter regions of the studied genes.

## Results

### Characteristics of study participants

One hundred sixteen BC patients and one hundred fourteen controls were enrolled in this study. Sociodemographic variables, potential BC risk factors of patients and controls, and clinical-histopathological characteristics of BC patients are presented in Table 1. The mean age for BC patients was 69.67 ± 11.26 and 66.71 ± 11.76 for controls. We showed a significant difference between the distribution of material status (free, married, widow/widower) and distribution of professional activity (physical work, mental work, unemployment, and pension) The blood analysis detected a significant difference between case and control for the level of RBC, HCT, HGB, RDW, WBC, glucose, creatinine, potassium (*p* < 0.05). In the case of urine analysis, there were significantly more subjects with positive protein and bilirubin occurrence for BC among the patients compared to controls (*p* < 0.001, *p* < 0.01, respectively). Similarly, urine samples of BC patients were more turbid than controls. Moreover, the urine of BC patients was characterised by an increased number of RBC, WBC, and bacteria for the power field as compared to healthy volunteers (*p* < 0.001, *p* < 0.01, *p* < 0.001, respectively).

**Table 1:** Socio-demographic and clinical characteristics of investigated subjects.

Feature			Controls (n = 114) Frequency	Patients with BC (n = 116) Frequency	p*
**Demographic characteristics of the study participants**					
Gender	Females		0.34	0.28	0.278
	Males		0.66	0.72	
**Age**	**Mean ± SD**		**66.71 ± 11.76**	**69.67 ± 11.26**	**0.031**
	**Range**		**28–91**	**20–92**	
Education	Primary (basic) education		0.16	0.30	0.087
	Vocational education		0.39	0.25	
	High school education		0.33	0.42	
	University degree		0.12	0.03	
Residency	Village		0.36	0.46	0.805
	A city with a population under 50 thou. Residents		0.39	0.16	
	A city with a population over 50 thou. Residents		0.25	0.39	
**Marital status**	**Free**		**0.25**	**0.13**	**0.003**
	**Married**		**0.67**	**0.70**	
	**Widow/widower**		**0.08**	**0.17**	
**Professional activity**	**Physical work**		**0.28**	**0.20**	**0.031**
	**Mental work**		**0.17**	**0.05**	
	**Unemployment**		**0.01**	**0.04**	
	**Pension**		**0.64**	**0.71**	
Smoking	Never		0.56	0.31	0.367
	Former		0.24	0.38	
	Current		0.20	0.31	
BMI [kg/m^2]^	Mean ± SD		27.69 ± 3.51	27.12 ± 4.83	0.305
	< 25		0.31	0.36	
	25–30		0.42	0.37	
	> 30		0.27	0.27	
Daily fluid intake	< 2 L/day		0.52	0.50	0.791
	> 2 L/day		0.48	0.50	
Daily coffee consumption (number of cups, one cup has a capacity of 200 mL)	0		0.21	0.27	0.369
	1		0.44	0.41	
	2–3		0.32	0.30	
	> 4		0.03	0.02	

Feature/parameters			Controls (n = 114) mean ± SD	Patients with BC (n = 116) mean ± SD	*p*
**Total blood count of the study participants**					
** Red Blood Cells**—**RBC (× 10**^**12**^**/L)**			**4.49 ± 0.61**	**4.29 ± 0.71**	**0.039**
** Haematocrit**—**HCT (%)**			**40.91 ± 5.26**	**38.80 ± 6.58**	**0.014**
** Haemoglobin**—**HGB (g/L)**			**13.56 ± 2.44**	**12.91 ± 2.33**	**0.030**
Mean corpuscular volume—MCV (fL)			91.25 ± 5.34	90.49 ± 5.97	0.135
Mean cell haemoglobin—MCH (pg/cell)			30.20 ± 3.20	30.24 ± 2.74	0.290
Mean corpuscular haemoglobin concentration—MCHC (g/L)			33.27 ± 1.22	33.11 ± 1.52	0.207
** Red cell distribution width**—**RDW (%)**			**13.50 ± 1.49**	**13.90 ± 1.32**	**0.010**
Haemoglobin distribution width—HDW (g/L)			2.53 ± 0.25	2.56 ± 0.40	0.467
** White blood cells**—**WBC (× 10**^**9**^**/L)**			**9.23 ± 18.67**	**8.88 ± 5.07**	**0.032**
%HYPO			2.34 ± 4.23	3.63 ± 7.10	0.157
%MIKRO			1.35 ± 2.76	1.32 ± 2.56	0.903
%MAKRO			1.44 ± 1.85	1.77 ± 4.05	0.978
%HYPER			0.73 ± 0.54	0.72 ± 0.85	0.091
Blood platelets—PLT (× 10^9^/L)			234.95 ± 70.80	259.25 ± 106.83	0.127
Mean platelet volume—MPV (fL)			8.68 ± 1.53	8.98 ± 1.04	0.081
**Blood biochemical parameters**					
** Glucose (mmol/L)**			**5.92 ± 2.16**	**6.64 ± 2.29**	** < 0.001**
** Creatinine (µmol/L)**			**106.94 ± 12.14**	**127.02 ± 156.58**	**0.005**
Sodium (mmol/L)			139.93 ± 2.75	139.48 ± 4.25	0.391
** Potassium (mmol/L)**			**5.57 ± 9.42**	**4.60 ± 0.56**	**0.043**
**Coagulation panel**					
Prothrombin time (s)			12.68 ± 2.48	12.19 ± 1.20	0.285
Prothrombin index (%)			92.90 ± 11.89	95.30 ± 8.59	0.237
International normalised ratio (INR)			1.13 ± 0.23	1.09 ± 0.11	0.883
Activated partial thromboplastin time (APTT, s)			30.67 ± 3.91	30.00 ± 3.11	0.609
Fibrinogen (mg/L)			400.24 ± 145.26	426.59 ± 178.41	0.362
**Dipstick urinalysis**					
pH			5.84 ± 0.88	5.89 ± 1.01	0.911
Specific gravity			1.02 ± 0.01	1.02 ± 0.01	0.842

Feature/parameters			Controls (n = 114) Frequency	Patients with BC (n = 116) Frequency	*p*
WBC	Absence per high-power field		0.68	0.45	0.077
	Single per high-power field		0.29	0.25	
	Numerous per high-power field		0.04	0.30	
Nitrite	Negative		0.88	0.88	0.658
	Positive		0.12	0.12	
Glucose	Negative		0.98	0.95	0.262
	Positive		0.02	0.05	
**Protein**	**Negative**		**0.76**	**0.44**	** < 0.001**
	**Positive**		**0.24**	**0.56**	
Ketones	Negative		0.90	0.90	0.964
	Positive		0.10	0.10	
**Bilirubin**	**Negative**		**0.98**	**0.89**	**0.006**
	**Positive**		**0.02**	**0.11**	
Urobilinogen	Normal level		1.00	0.94	0.711
	Above normal		0.00	0.06	
Colour	Pale yellow		0.05	0.11	0.218
	Straw/yellow		0.82	0.63	
	Dark yellow		0.02	0.02	
	Amber		0.05	0.10	
	Brown		0.01	0.06	
	Red		0.04	0.08	
**Clarity**	**Clear**		**0.74**	**0.48**	** < 0.001**
	**Slightly cloudy**		**0.12**	**0.25**	
	**Cloudy**		**0.06**	**0.07**	
	**Very cloudy**		**0.08**	**0.20**	
**Urine microscopy**					
** RBC**	**0–3/high power field**		**0.78**	**0.33**	** < 0.001**
	**3–5/high power field**		**0.07**	**0.03**	
	**5–10//high power field**		**0.03**	**0.12**	
	**10–15/high power field**		**0.03**	**0.22**	
	**15–20/high power field**		**0.03**	**0.04**	
	**20–25/high power field**		**0.06**	**0.26**	
** WBC**	**1–3/high power field**		**0.66**	**0.45**	**0.006**
	**3–5/high power field**		**0.05**	**0.14**	
	**5–10/high power field**		**0.19**	**0.20**	
	**10–15/high power field**		**0.01**	**0.05**	
	**20–25/high power field**		**0.08**	**0.15**	
Squamous epithelial cells	Single per high power field		0.82	0.69	0.177
	Sparse per high power field		0.10	0.23	
	Many per high power field		0.08	0.08	
Mucus thread	Single per high power field		0.00	0.38	0.851
	Sparse per high power field		0.80	0.24	
	Many per high power field		0.20	0.38	
** Bacteria**	**Lack of/single per high power field**		**0.65**	**0.01**	** < 0.001**
	**Sparse per high power field**		**0.27**	**0.39**	
	**Many per high power field**		**0.08**	**0.60**	

Feature/parameters			Controls (n = 114) Frequency	Patients with BC (n = 116) Frequency	*p*
**Additional information**					
Comorbidities	**Hypertension**	**Yes**	**0.53**	**0.31**	** < 0.001**
		**No**	**0.47**	**0.69**	
	Diabetes	Yes	0.17	0.16	0.955
		No	0.83	0.84	
	**Hypercholesterolaemia**	**Yes**	**0.22**	**0.10**	**0.017**
		**No**	**0.78**	**0.90**	
The presence in the family history of bladder cancer	Yes		n/d	0.10	n/d
	No		n/d	0.90	
Actually therapy for bladder cancer	Surgical treatment—TURBT (transurethral resection of bladder tumour)		n/d	0.91	n/d
	Chemotherapy		n/d	0.03	
	Cystectomy		n/d	0.06	
**Symptoms accompanying BC**					
Haematuria with clots	Yes		n/d	0.68	n/d
	No		n/d	0.32	
Dysuria	Yes		n/d	0.65	n/d
	No		n/d	0.35	
Recurrent urinary tract infections	Yes		n/d	0.63	n/d
	No		n/d	0.37	
Pollakiuria	Yes		n/d	0.61	n/d
	No		n/d	0.39	
Urgent pressures	Yes		n/d	0.68	n/d
	No		n/d	0.32	
The feeling of being left behind after voiding	Yes		n/d	0.08	n/d
	No		n/d	0.92	
Waiting for micturition	Yes		n/d	0.05	n/d
	No		n/d	0.95	
Urinary incontinence problem	Yes		n/d	0.54	n/d
	No		n/d	0.46	
Lower abdominal pain	Yes		n/d	0.51	n/d
	No		n/d	0.49	
Weight loss	Yes		n/d	0.41 0.59	n/d
	No		n/d		
Zubrod fitness level (ECOG scale)	0		n/d	0.84	n/d
	1		n/d	0.09	
	2		n/d	0.02	
	3		n/d	0.04	
	4		n/d	0.01	
	5		n/d	0	
TNM Classification of Malignant Tumors (TNM)	Tx		n/d	0.01	n/d
	T0		n/d	0.01	
	Ta		n/d	0.38	
	Tis		n/d	0.01	
	T1		n/d	0.41	
	T2		n/d	0.17	
	T3		n/d	0.01	
	T4		n/d	0.00	
Status of regional lymph nodes	N0–N1		n/d	0.82	n/d
	≥ N2		n/d	0.18	
The presence of distant metastases	M0		n/d	0.82	n/d
	M1		n/d	0.18	
Pathomorphology of nonmuscle-invasive tumors	Urothelial papilloma		n/d	0.02	n/d
	Inverted papilloma		n/d	0.00	
	Papillary urothelial neoplasm of low malignant potential (PUN-LMP)		n/d	0.16	
	Low-grade papillary urothelial carcinoma		n/d	0.42	
	High-grade papillary urothelial Carcinoma		n/d	0.35	
Pathomorphology of tumors infiltrating the muscle membrane	Invasive urothelial carcinoma		n/d	0.00	n/d
	Squamous cell carcinoma		n/d	0.04	
	Glandular carcinoma		n/d	0.00	
	Small cell carcinoma		n/d	0.00	
	Undifferentiated carcinoma		n/d	0.00	
	Other		n/d	0.02	

*p < 0.05 are in bold.

### Single nucleotide polymorphisms of the SOD2 and NOS2 as the risk of BC occurrence

To perform genotype and allele distribution analysis, BC patients and controls were divided into groups corresponding to three genotypes and two alleles for each studied SNP, and obtained results are presented in Table 2. Among analysed polymorphisms, only heterozygote of the g.-1026 C > A—*NOS2* (rs2779249) SNP was associated with a decreased risk of BC development (*p* < 0.05). In the case of c. 47 C > T—*SOD2* (rs4880) and c.1823 C > T (p. Ser608Leu)—*NOS2* (rs2297518) polymorphisms, we did not detect any correlation (*p* > 0.05) between genotypes/alleles of these SNPs and BC occurrence.

**Table 2:** Distribution of genotypes and alleles of the c. 47 C > T—*SOD2* (rs4880) and c.1823 C > T (p. Ser608Leu)—*NOS2* (rs2297518) as well as g.-1026 C > A—*NOS2* (rs2779249) and odds ratios (ORs) with confidence intervals (95% CIs) in BC patients and controls.

Genotype/Allele	Control (n = 114)		BC (n = 116)		Crude OR (95% CI)*	*p*	Adjusted OR (95% CI)*	*p*
	Number	Frequency	Number	Frequency				
c. 47 C > T (p.Val16Ala)—*SOD2* (rs4880)								
Frequencies								
C/C	29	0.254	21	0.181	0.648 (0.344–1.221)	0.179	0.654 (0.347–1.235)	0.191
C/T	56	0.491	66	0.569	1.275 (0.759–2.141)	0.359	1.283 (0.763–2.158)	0.347
T/T	29	0.254	29	0.250	1.022 (0.566–1.848)	0.941	1.007 (0.556–1.823)	0.983
	χ^2^ = 230.001; *p* = 0.432							
C	114	0.500	108	0.466	0.834 (0.573–1.215)	0.344	0.843 (0.578–1.229)	0.374
T	114	0.500	124	0.534	1.156 (0.794–1.682)	0.499	1.146 (0.786–1.669)	0.479
Carriage rates								
C ( +)	85	0.746	87	0.750	1.024 (0.564–1.856)	0.939	1.016 (0.559–1.845)	0.959
C (−)	29	0.254	21	0.181	0.648 (0.344–1.221)	0.179	0.668 (0.353–1.263)	0.215
T ( +)	85	0.746	95	0.819	1.543 (0.819–2.907)	0.179	1.497 (0.792–2.832)	0.215
T (−)	29	0.254	29	0.250	0.977 (0.539–1.772)	0.939	0.985 (0.542–1.789)	0.859
c.1823 C > T (p. Ser608Leu)—*NOS2* (rs2297518)								
Frequencies								
C/C	76	0.667	82	0.707	1.206 (0.690–2.107)	0.511	1.194 (0.682–2.090)	0.535
C/T	35	0.307	32	0.276	0.860 (0.487–1.519)	0.603	0.867 (0.490–1.534)	0.623
T/T	3	0.026	2	0.017	0.649 (0.106–3.959)	0.639	0.666 (0.109–4.087)	0.661
	χ^2^ = 229.983; *p* = 0.414							
C	187	0.820	196	0.845	1.204 (0.728–1.990)	0.469	1.192 (0.720–1.973)	0.495
T	41	0.180	36	0.155	0.831 (0.502–1.373)	0.469	0.839 (0.507–1.389)	0.495
Carriage rates								
C (+)	111	0.974	114	0.983	1.541 (0.253–9.396)	0.639	1.500 (0.245–9.201)	0.661
C (−)	3	0.026	2	0.017	1.206 (0.690–2.107)	0.511	1.194 (0.682–2.090)	0.535
T (+)	38	0.333	34	0.293	0.829 (0.475–1.449)	0.511	0.838 (0.479–1.466)	0.535
T (−)	76	0.667	82	0.707	0.649 (0.106–3.959)	0.639	0.666 (0.109–4.087)	0.661
g.-1026 C > A—*NOS2* (rs2779249)								
Frequencies								
C/C	57	0.500	65	0.560	1.275 (0.759–2.141)	0.360	1.240 (0.735–2.091)	0.420
**C/A**	**51**	**0.447**	**37**	**0.319**	**0.579 (0.338–0.990)**	**0.046**	**0.593 (0.346–0.995)**	**0.049**
A/A	6	0.053	14	0.121	2.471 (0.914–6.675)	0.074	2.474 (0.914–6.701)	0.075
	χ^2^ = 230.000; *p* = 0.432							
C	165	0.724	167	0.720	0.982 (0.659–1.463)	0.928	0.964 (0.646–1.440)	0.859
A	63	0.276	65	0.280	1.019 (0.684–1.517)	0.928	1.037 (0.694–1.549)	0.859
Carriage rates								
C (+)	108	0.947	102	0.879	0.405 (0.150–1.094)	0.074	0.404 (0.149–1.094)	0.075
C (−)	57	0.500	65	0.560	1.275 (0.759–2.141)	0.360	1.240 (0.735–2.091)	0.420
A (+)	57	0.500	51	0.440	0.785 (0.467–1.318)	0.360	0.806 (0.478–1.360)	0.420
A (−)	6	0.053	14	0.121	2.471 (0.914–6.675)	0.074	2.474 (0.915–6.701)	0.075

*Crude OR means OR calculated with conventional logistic regression; for the significant outcomes, adjusted OR means OR calculated with conventional logistic regression adjusted sex; for the significant outcomes,* p* < 0.05 along with the corresponding ORs are in bold (for the genotypes/alleles with a protective effect).

### Association between combined genotypes of SOD2 and NOS2 SNPs and the risk of the BC development—gene–gene interaction

We also investigated the link between BC occurrence and combined genotypes of studied SNPs. The distribution of combined genotypes of the c. 47C > T (rs4880) in the *SOD2* gene, c.1823 C > T (p. Ser608Leu) (rs2297518) and g.-1026 C > A (rs2779249) in *NOS2* gene polymorphisms for cancer patients and controls is shown in Supplementary Table 1. Unfortunately, we did not find any association between combined genotypes of analysed polymorphisms and the BC occurrence (*p* > 0.05). Moreover, additional synergy factor (SF) analysis (Supplementary Table 2) by Mario Cortina-Borja et al. (2009) recommendations’^35^ did not also confirm any interactions between studied polymorphisms (*p* > 0.05).

### Haplotypes and the risk of BC occurrence

**Figure 1:**
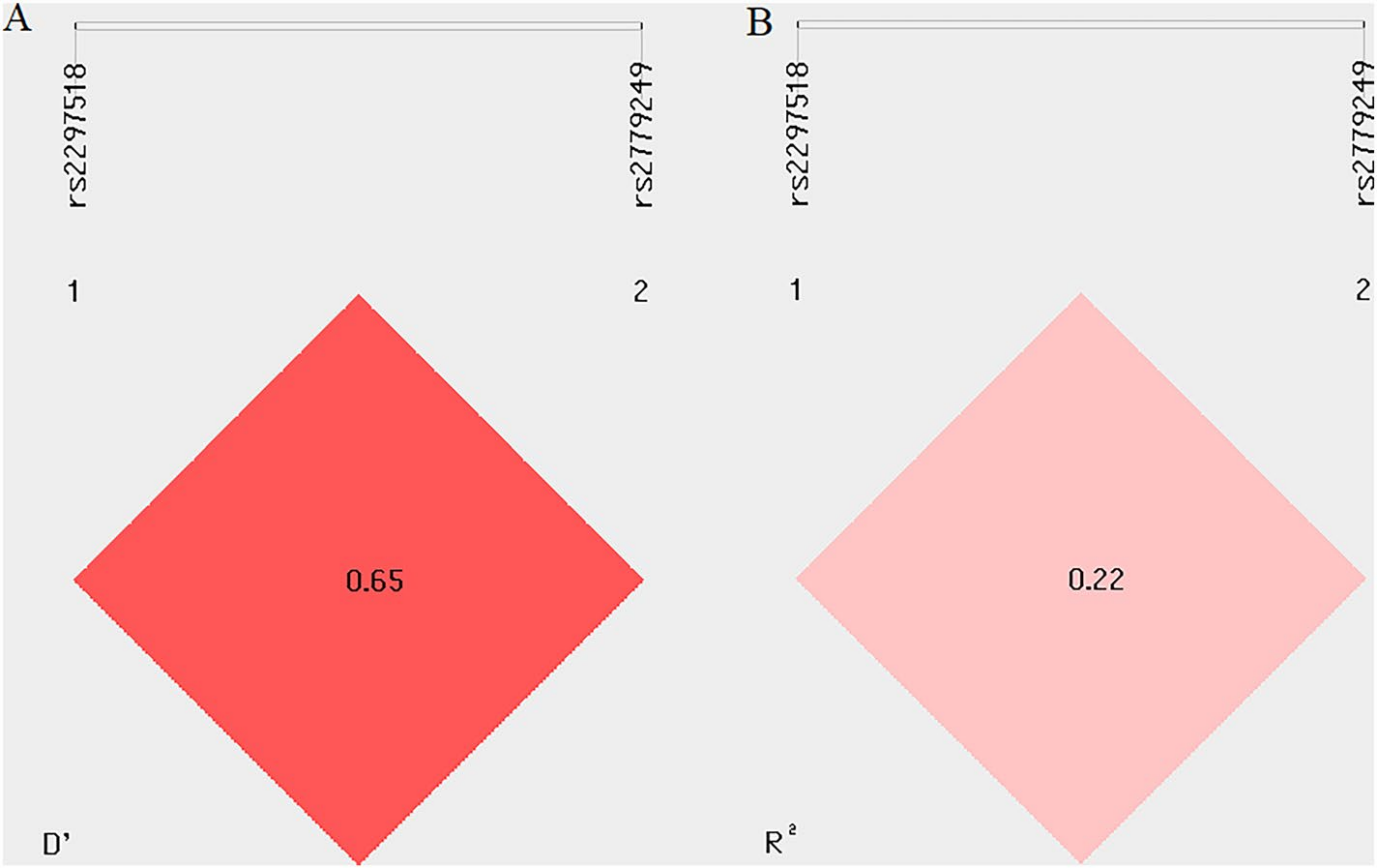
LD analysis of rs2297518 and 2,779,249 polymorphisms in the *NOS2* gene. Pairwise D’ values (**A**). Pairwise R^2^ values (**B**). R^2^ ≥ 0.8—high LD.

In this study, we also checked the association between haplotypes of the c.1823 C > T and the g.-1026 C > A SNPs of the *NOS2* gene and BC occurrence. LD analysis^36–39^ revealed that among analysed SNPs in the *NOS2* gene, we identified no studied polymorphisms as strong linkage disequilibrium regions in *NOS2* (R^2^ < 0.8) (Fig. 1.). Supplementary Table 3 shows the distribution of such haplotypes. Unfortunately, our analysis showed no significant link (*p* > 0.05) between the haplotypes and BC development.

### The association between studied polymorphisms and clinical-histopathological characteristics of BC patients

We checked the association between *SOD2* and *NOS2* polymorphisms for BC patients stratified by TNM staging^40^ and the World Health Organization/International Society of Urological Pathology (WHO/ISUP) grading system^41^. For this, we divided group of patients with BC into subgroups according to the size of the primary tumour (Ta, T1, ≥ T2), the status of lymph nodes (N0, ≥ N1), and the distant metastasis (M0, M1) according to the TNM Classification of Malignant Tumours, 8^th^ Edition developed by the Union for International Cancer Control (UICC). In turn, in the case of the World Health Organization/International Society of Urological Pathology (WHO/ISUP) classification system, because the number of BC subjects with urothelial and inverted papilloma as well as tumours infiltrating the muscle membrane was limited to a few, these cases were omitted from the analysis and patients were divided into papillary urothelial neoplasm of low malignant potential (PUN-LMP), low-grade papillary urothelial carcinoma and high-grade papillary urothelial carcinoma. However, we did not find any association between the c. 47C > T, c.1823 C > T (p. Ser608Leu) and g.-1026 C > A polymorphisms and TNM stage as well as WHO/ISUP tumour grade (Supplementary Table 4).

### SNPs of SOD2 and NOS2, and BC occurrence in the male and female subpopulation

Previous epidemiological analyses show that men are at a higher risk of developing BC than women^4^. Therefore, we divided the control group and patients with BC into female and male subgroups and analysed the distribution of genotypes and alleles of studied polymorphisms in men and women with BC. Interestingly, our results confirmed that polymorphic variants might modulate the risk of BC occurrence depending on gender (Table 3). We detected that heterozygotes of c.1823 C > T (p. Ser608Leu) (rs2297518) and g.-1026 C > A (rs2779249) in the *NOS2* gene polymorphisms were associated with a reduced risk of BC development in an only female subpopulation (*p* < 0.05, *p* < 0.01, respectively). Moreover, C/C homozygotes of g.-1026 C > A (rs2779249) SNP were associated with increased occurrence of BC in women, while in the male subpopulation, we did not observe this correlation (*p* < 0.05).

**Table 3:** Distribution of genotypes and alleles of the c. 47 C > T (p.Val16Ala)—*SOD2* (rs4880), c.1823 C > T (p. Ser608Leu)—*NOS2* (rs2297518) and g.-1026 C > A—*NOS2* (rs2779249) and ORs with 95% CIs in men and women with BC.

Genotype/Allele	WOMEN (n = 71)				MEN (n = 159)			
	Control (n = 39)	BC (n = 32)	Crude OR (95% CI)*	*p*	Control (n = 75)	BC (n = 84)	Crude OR (95% CI)*	*p*
	N (Freq.)	N (Freq.)			N (Freq.)	N (Freq.)		
c. 47 C > T (p.Val16Ala)—*SOD2* (rs4880)								
T/T	12 (0.308)	8 (0.250)	0.750 (0.262–2.143)	0.591	17 (0.227)	13 (0.155)	0.625 (0.280–1.392)	0.250
C/T	17 (0.436)	15 (0.469)	1.142 (0.446–2.922)	0.782	39 (0.520)	39 (0.607)	1.427 (0.760–2.679)	0.269
C/C	10 (0.256)	9 (0.281)	1.135 (0.396–3.255)	0.814	19 (0.253)	20 (0.238)	0.921 (0.447–1.898)	0.824
	χ^2^ = 70.997; *p* = 0.378				χ^2^ = 159.007; *p* = 0.418			
C	41 (0.526)	31 (0.484)	1.162 (0.618–2.188)	0.641	73 (0.487)	77 (0.458)	0.877 (0.545–1.410)	0.587
T	37 (0.474)	33 (0.516)	0.860 (0.457–1.619)	0.641	77 (0.513)	91 (0.542)	1.141 (0.709–1.835)	0.587
c.1823 C > T (p. Ser608Leu)—*NOS2* (rs2297518)								
C/C	22 (0.564)	25 (0.781)	2.760 (0.965–7.888)	0.058	54 (0.720)	57 (0.679)	0.821 (0.416–1.622)	0.570
**T/C**	**16 (0.410)**	**6 (0.188)**	**0.332 (0.111–0.990)**	**0.048**	19 (0.253)	26 (0.310)	1.321 (0.659–2.651)	0.433
T/T	1 (0.026)	1 (0.031)	1.226 (0.074–20.403)	0.887	2 (0.027)	1 (0.012)	0.440 (0.039–4.950)	0.506
	χ^2^ = 71.623; *p* = 0.359				χ^2^ = 159.003; *p* = 0.418			
C	60 (0.769)	56 (0.875)	2.184 (0.849–5.622)	0.105	127 (0.847)	140 (0.833)	0.901 (0.486–1.670)	0.740
T	18 (0.231)	8 (0.125)	0.458 (0.178–1.178)	0.105	23 (0.153)	28 (0.167)	1.110 (0.599–2.059)	0.740
g.-1026 C > A—*NOS2* (rs2779249)								
* C/C*	*13 (0.333)*	*19 (0.594)*	*2.923 (1.108–7.711)*	*0.030*	44 (0.587)	46 (0.548)	0.853 (0.455–1.600)	0.620
**C/A**	**24 (0.615)**	**9 (0.281)**	**0.245 (0.090–0.668)**	**0.006**	27 (0.360)	28 (0.333)	0.889 (0.462–1.710)	0.724
A/A	2 (0.051)	4 (0.125)	2.643 (0.452–15.469)	0.281	4 (0.053)	10 (0.119)	2.399 (0.719–7.999)	0.155
	χ^2^ = 71.396; *p* = 0.366				χ^2^ = 158.961; *p* = 0.419			
C	50 (0.641)	47 (0.734)	1.611 (0.752–3.453)	0.220	115 (0.767)	120 (0.714)	0.780 (0.481–1.265)	0.313
A	28 (0.359)	17 (0.266)	0.621 (0.290–1.331)	0.220	35 (0.233)	48 (0.286)	1.282 (0.791–2.079)	0.313

*Crude OR means OR calculated with conventional logistic regression; for the significant outcomes, *p* < 0.05 along with the corresponding ORs are in italic (for the genotypes/alleles increasing the risk of BC) or in bold (for the genotypes/alleles with a protective effect).

### SNPs of genes encoding SOD2 and NOS2, and BC occurrence in groups with normal body weight/overweight and obesity and in the non-smoker/smoker subpopulation

In addition to gender, among the risk factors for the development of BC, cigarette smoking and excessive body weight (BMI above the norm) are also mentioned^4,10^. Thus, in our study, we performed the analysis of the distribution of genotypes and alleles of c. 47 C > T (p.Val16Ala) (rs4880), c.1823 C > T (p. Ser608Leu) (rs2297518) and g.-1026 C > A (rs2779249) polymorphisms in patients and controls in non-smoker/smoker subpopulations and subgroups with normal body weight/overweight and obesity. Our findings confirmed that polymorphic variants might modulate BC risk depending on the smoking (Table 4). We detected that the C/A genotype of g.-1026 C > A (rs2779249) polymorphism had a protective effect in the non-smoker group (*p* < 0.05). In the contrast, no this association was observed in the cigarette smoker subgroup (*p* < 0.05). In the case of BMI and polymorphism analysis, no association (*p* > 0.05) between genotypes/alleles of studied SNPs and BC occurrence was found in subgroups with normal body weight and overweight/obesity (Supplementary Table 5).

**Table 4:** Distribution of genotypes and alleles of the c. 47 C > T (p.Val16Ala)—*SOD2* (rs4880), c.1823 C > T (p. Ser608Leu)—*NOS2* (rs2297518) and g.-1026 C > A—*NOS2* (rs2779249) and ORs with 95% CIs in non-smokers and smokers with BC.

Genotype/Allele	NON-SMOKER (n = 104)				SMOKER (n = 126)			
	Control (n = 68)	BC (n = 36)	Crude OR (95% CI)*	*p*	Control (n = 46)	BC (n = 80)	Crude OR (95% CI)*	*p*
	N (Freq.)	N (Freq.)			N (Freq.)	N (Freq.)		
c.47 T > C (p.Val16Ala)—*SOD2* (rs4880)								
C/C	17 (0.250)	11 (0.306)	1.320 (0.538–3.236)	0.544	12 (0.261)	15 (0.188)	0.823 (0.355–1.909)	0.649
T/C	35 (0.515)	19 (0.528)	1.054 (0.469–2.366)	0.899	21 (0.457)	47 (0.588)	1.696 (0.816–3.523)	0.157
T/T	16 (0.235)	6 (0.167)	0.650 (0.230–1.840)	0.417	13 (0.283)	15 (0.188)	0.586 (0.250–1.374)	0.219
	χ^2^ = 103.973; *p* = 0.400				χ^2^ = 126.037; *p* = 0.407			
C	69 (0.507)	41 (0.569)	1.300 (0.720–2.347)	0.385	45 (0.489)	83 (0.519)	1.138 (0.666–1.944)	0.637
T	67 (0.493)	31 (0.431)	0.769 (0.426–1.389)	0.385	47 (0.511)	77 (0.481)	0.879 (0.514–1.502)	0.637
c.1823 C > T (p. Ser608Leu)—*NOS2* (rs2297518)								
C/C	46 (0.676)	27 (0.750)	1.435 (0.578–3.563)	0.437	30 (0.652)	55 (0.688)	1.173 (0.544–2.532)	0.163
C/T	20 (0.294)	9 (0.250)	0.800 (0.320–2.002)	0.633	15 (0.326)	23 (0.288)	0.834 (0.381–1.826)	0.650
T/T	2 (0.029)	0 (0.000)	0.000 (0.000- + inf.)	0.995	1 (0.022)	2 (0.025)	1.154 (0.102–13.085)	0.908
	χ^2^ = 103.839; *p* = 0.403				χ^2^ = 125.997; *p* = 0.408			
C	112 (0.824)	63 (0.824)	1.530 (0.655–3.569)	0.326	75 (0.815)	133 (0.831)	1.123 (0.565–2.230)	0.741
T	24 (0.176)	9 (0.125)	0.654 (0.280–1.526)	0.326	17 (0.185)	27 (0.169)	0.891 (0.448–1.770)	0.741
g.-1026 C > A—*NOS2* (rs2779249)								
C/C	30 (0.441)	22 (0.611)	1.990 (0.874–4.535)	0.101	27 (0.587)	43 (0.538)	0.818 (0.393–1.703)	0.591
**C/A**	**34 (0.500)**	**9 (0.250)**	**0.333 (0.137–0.813)**	**0.016**	17 (0.370)	28 (0.350)	0.919 (0.432–1.954)	0.825
A/A	4 (0.059)	5 (0.139)	2.581 (0.647–10.288)	0.179	2 (0.043)	9 (0.113)	2.789 (0.576–13.507)	0.203
	χ^2^ = 104.193; *p* = 0.394				χ^2^ = 125.875; *p* = 0.411			
C	94 (0.691)	53 (0.736)	1.246 (0.658–2.362)	0.500	71 (0.772)	114 (0.713)	0.750 (0.422–1.333)	0.327
A	42 (0.309)	19 (0.264)	0.802 (0.423–1.521)	0.500	21 (0.228)	46 (0.288)	1.333 (0.750–2.370)	0.327

*Crude OR means OR calculated with conventional logistic regression; for the significant outcomes, *p* < 0.05 along with the corresponding ORs are in bold (for the genotypes/alleles with a protective effect).

### SOD2 and NOS2 mRNA level analysis

**Figure 2:**
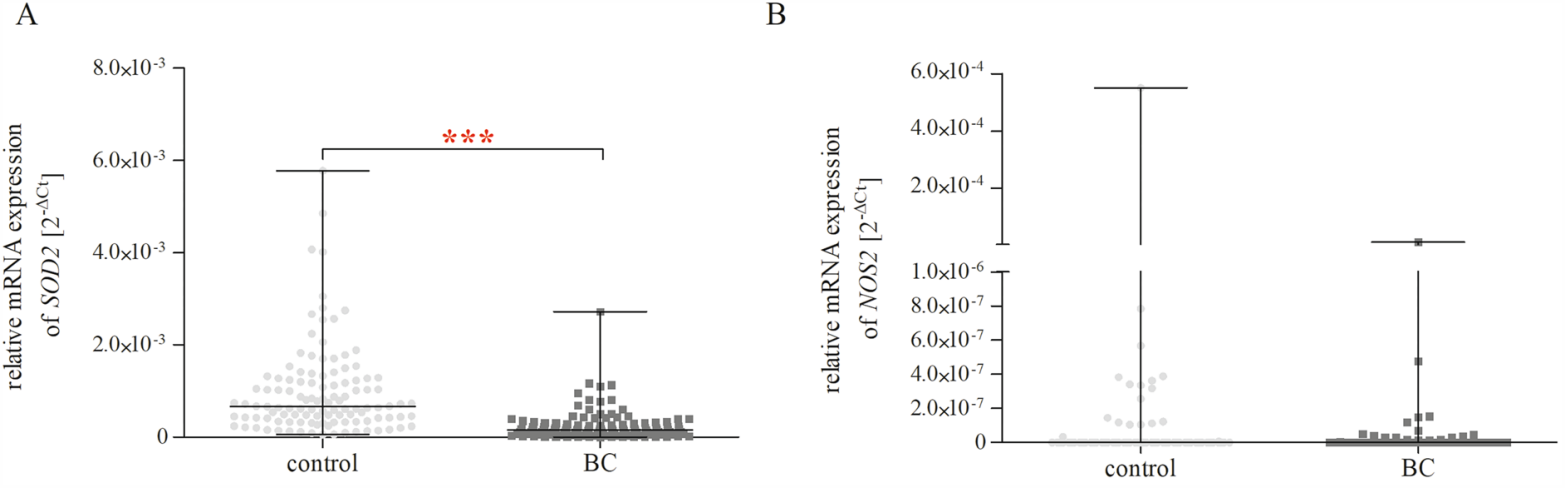
Relative mRNA expression of *SOD2* (**A**) and *NOS2* (**B**) genes of controls (n controls = 114) and patients with BC (n patients with BC = 116). Relative gene expression levels were calculated by the 2^−ΔCt^ method (C_t gene_− C_t 18S_) method. The data are plotted as individual values and the median with interquartile range is indicated by the horizontal bars; ***p < 0.001.

The presented study also included expression analysis at the mRNA level of *SOD2* and *NOS2*, and obtained results have been presented in Fig. 2. We found that patients with BC were characterised by lower *SOD2* expression as compared to controls (*p* < 0.001). In the case of the *NOS2* expression analysis, no significant difference was found between patients with BC and the control group (*p* > 0.05).

### SOD2 and NOS2 expression and correlation with clinical-histopathological parameters

Similar to polymorphisms, we checked the association between *SOD2* and *NOS2* expression for BC patients stratified by TNM staging^40^ and WHO/ISUP grading system^41^. Unfortunately, in the case of both *SOD2* and *NOS2* expression levels, we detected no statistical differences (*p* > 0.05) between TNM staging subgroups and between pathomorphological subgroups (Supplementary Figure 1).

### SOD2 and NOS2 expression in the genotype subgroups

**Figure 3:**
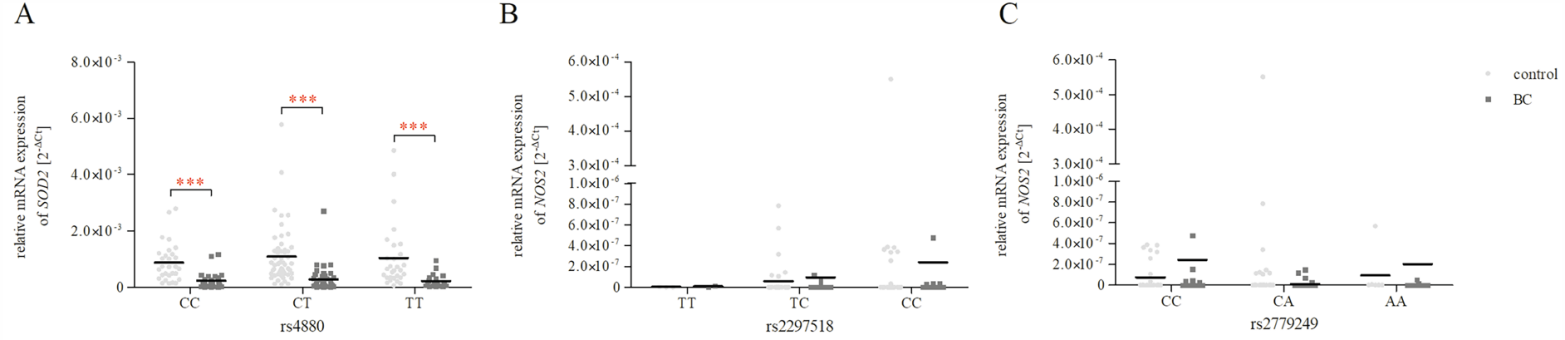
Relative *SOD2* (**A**) and *NOS2* (**B**, **C**) expression in the genotype groups of all studied SNPs, expressed as the 2^−ΔCt^ (C_t gene_− C_t 18S_) method for each sample. The data are plotted as individual values and the median with interquartile range is indicated by the horizontal bars; ***p < 0.001.

SNPs may modulate gene function as well as the occurrence of phenotypic differences. SNPs may lead to the change of encoded amino acids (non-synonymous), or may be silent (synonymous), or simply present in non-coding regions. They can affect promoter activity (gene expression), messenger RNA (mRNA) conformation (stability), and subcellular localisation of mRNA and/or proteins, and therefore can cause disease^42^. Therefore, to evaluate whether the studied polymorphisms may impact the mRNA expression of *SOD2* and *NOS2*, the patients were divided according to genotype, and the gene expression was compared. Unfortunately, we found no impact genotypes of each analysed polymorphism on *SOD2* and *NOS2* expression (Supplementary Figure 2). Moreover, we also checked the existence of significant differences in *SOD2* and *NOS2* expression between the control group and patients with BC in the genotype groups (Fig. 3). In the case of all genotypes of c.47 C > T (rs4880) *SOD2* gene, patients with BC were characterised by lower *SOD2* expression than the control group (*p* < 0.001). In the case of both polymorphisms of the *NOS2* gene, there were no significant differences between the studied groups for each genotype (*p* > 0.05).

### Effect of gender/BMI/cigarette smoking and BC on the mRNA expression of SOD2 and NOS2

**Figure 4:**
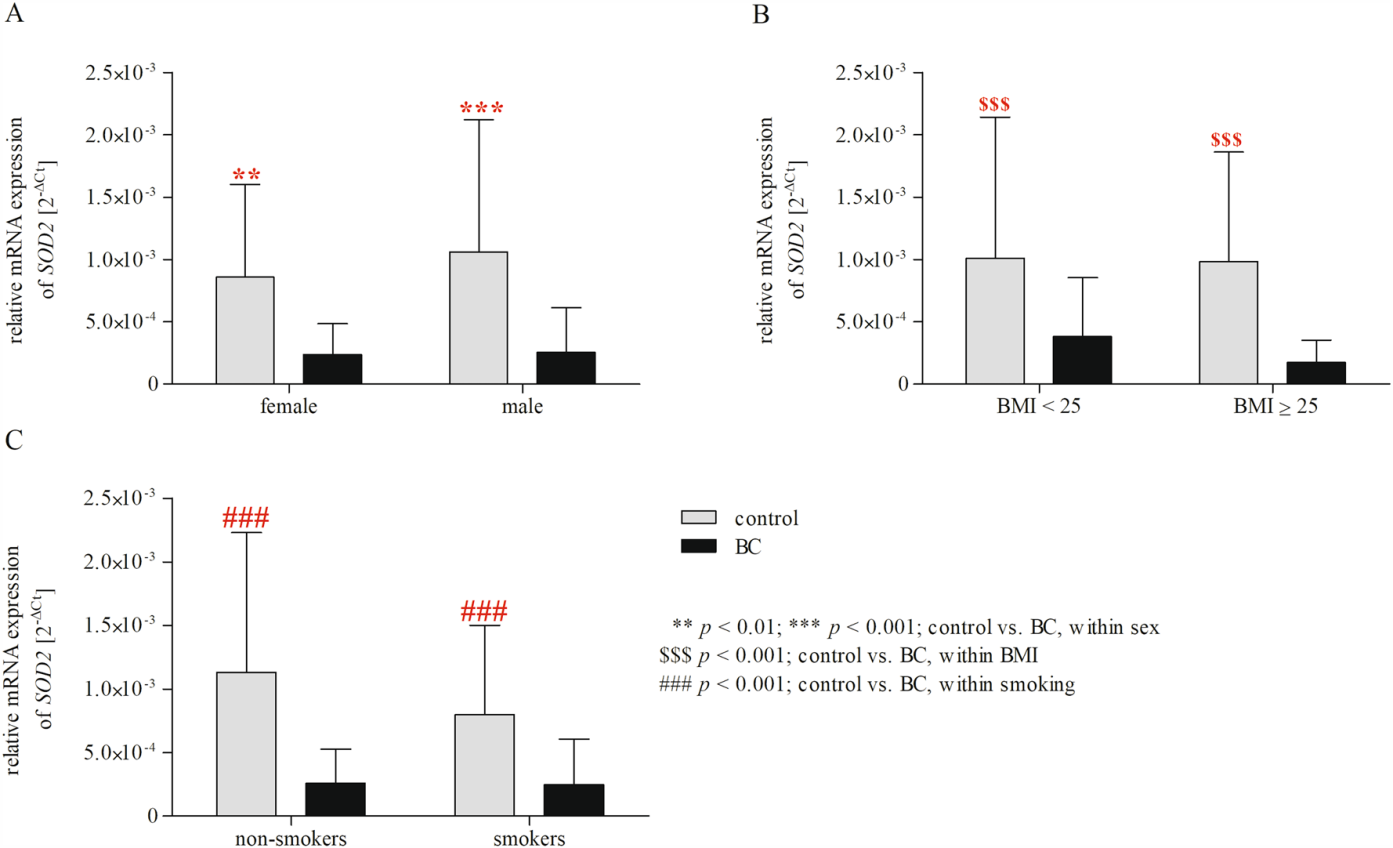
Two-way ANOVA with Bonferroni post hoc test shows significant effects of gender (**A**), BMI (**B**), cigarette smoking (**C**), and BC on the mRNA expression of *SOD2*. Gene expression in PBMCs is expressed as the 2^−ΔCt^ (C_t gene _− C_t 18S_) method. The data are presented as mean ± SD.

As mentioned before, gender, smoking, and high BMI increase the risk of BC development^4,10^. Therefore, we checked the existence of significant differences in *SOD2* and *NOS2* expression between the female and male population; smokers and non-smokers; subjects with BMI < 25, and subjects with BMI ≥ 25 (Table 5, Fig. 4). We found that *SOD2* expression was higher in the control group than in patients with BC in female (*p* < 0.01) and male (*p* < 0.001) populations, BMI < 25 (*p* < 0.001) and BMI ≥ 25 (*p* < 0.001) groups, non-smokers (*p* < 0.001) and smokers (*p* < 0.001). However two-way ANOVA analysis did not show significant effects interaction of gender/BMI/cigarette smoking × group for *NOS2* expression (*p* > 0.05). In the cases of gender, BMI, and cigarette smoking, we observed no impact on *NOS2* expression (Table 5, Supplementary Figure 3).

**Table 5:** Results of two-way ANOVA analyses on mRNA expression of *SOD2* and *NOS2*.

Factor	Gene	Study groups	Gender/BMI/Smoking	Interaction			
		F	*p*	F	*p*	F	p*
Gender	***SOD2***	**47.300**	** < 0.001**	1.166	0.281	0.804	0.371
	*NOS2*	1.834	0.177	1.805	0.180	1.902	0.169
BMI	***SOD2***	**51.611**	** < 0.001**	1.367	0.244	0.757	0.385
	*NOS2*	0.517	0.473	0.566	0.453	0.508	0.477
Smoking	***SOD2***	**50.719**	** < 0.001**	2.845	0.093	2.580	0.110
	*NOS2*	1.401	0.238	1.476	0.226	1.397	0.238

*p < 0.05 are in bold.

### The methylation status of SOD2 and NOS2 promoter regions

**Figure 5:**
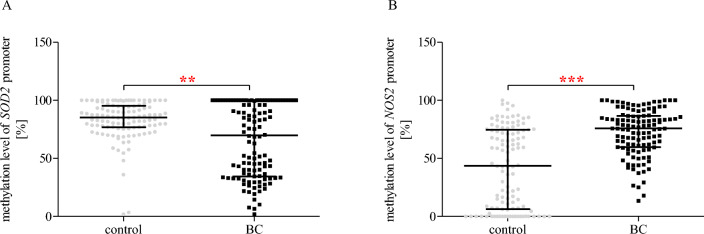
Methylation status of *SOD2* (**A**) and *NOS2* (**B**) promoters in controls and BC patients. The data are plotted as individual values and the median with interquartile range is indicated by the horizontal bars; **p < 0.01; ***p < 0.001.

Our study also included an analysis of the methylation status of *SOD2* and *NOS2* promoter regions, and obtained results have been presented in Fig. 5. We found that the patients with BC were characterised by the lower (*p* < 0.01) methylation level of the *SOD2* promoter region (Fig. 5A) and higher *NOS2* promoter methylation (*p* < 0.001) compared to the controls (Fig. 5B).

### Methylation of SOD2 and NOS2 promoter region and correlation with size or direct extent of the primary tumour, status of lymph nodes metastasis, and distant metastasis according to the TNM classification as well as the grading of histological malignancy

We also checked the impact of primary tumour size, the status of lymph nodes metastasis and distant metastasis, as well as the grading of histological malignancy on the level of methylation of *SOD2* and *NOS2* promoter regions (Supplementary Figure 4). For this purpose, similarly to the expression level analyses, we divided them into appropriate subgroups according to TNM staging^40^ and WHO/ISUP grading system^41^. Unfortunately, we detected no statistical differences (*p* > 0.05) between TNM staging subgroups and between pathomorphological subgroups in the case of methylation levels of *SOD2* and *NOS2* promoters.

### Effect of gender/BMI/cigarette smoking and BC on the methylation level of SOD2 and NOS2 promoter regions

**Figure 6:**
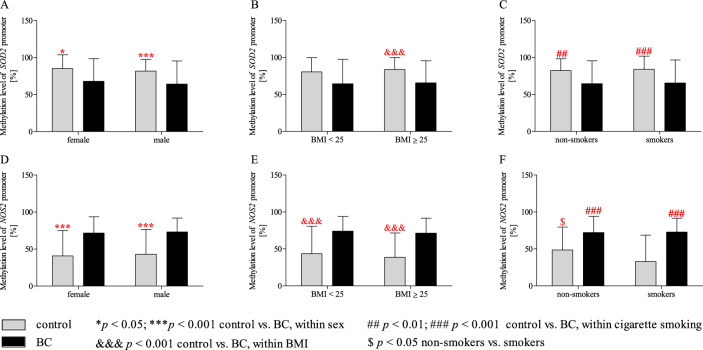
Two-way ANOVA with Bonferroni post hoc test shows significant effects of gender (**A**, **D**), BMI (**B**, **E**), cigarette smoking (**C**, **F**), and BC on the methylation status of *SOD2* and *NOS2* promoter region. The data are presented as mean ± SD.

Statistical analysis using two-way ANOVA (Table 6), which showed a difference between control and BC (*p* < 0.001) in all studied genes, also indicates a significant effect of cigarette smoking for *NOS2* promoter methylation status (*p* < 0.05). Additionally, two-way ANOVA analysis showed significant effects interaction of cigarette smoking × group for methylation level of *NOS2* promoter region (*p* < 0.05). Two-way ANOVA with Bonferroni post hoc test (Fig. 6) showed that patients with BC were characterised by significantly reduced methylation levels of *SOD2* promoter compared to the control group in women and men subgroups (*p* < 0.05 and *p* < 0.001, respectively) (Fig. 6A). Moreover, we found a decreased methylation status of *SOD2* in patients with BC compared to the control group only in a subgroup with BMI ≥ 25 (Fig. 6B). In turn, *SOD2* methylation level was lower in BC patients than in controls among smoker and non-smoker groups (*p* < 0.01 and *p* < 0.001, respectively) (Fig. 6C). In the case of *NOS2* promoter, methylation status was higher in BC patients than in controls in both women and men populations as well as BMI < 25 and BMI ≥ BMI groups (*p* < 0.001) (Fig. 6D,E). Moreover, statistical analysis detected that the methylation level of the *NOS2* promoter was significantly higher in patients with BC than in controls among non-smokers and smokers (*p* < 0.001) (Fig. 6F). Interestingly, patients with BC were characterised by the significantly elevated methylation status of *NOS2* promoter compared to the control group only in a subgroup of non-smokers (*p* < 0.05). This relationship was not observed in women (Fig. 6F).

**Table 6:** Results of two-way ANOVA analyses on the methylation status of *SOD2* and *NOS2* promoters.

Factor	Gene	Study groups	Gender/BMI/smoking	Interaction			
		F	*p*	F	*p*	F	p*
Gender	*SOD2*	**23.282**	** < 0.001**	0.960	0.328	0.002	0.974
	*NOS2*	**57.454**	** < 0.001**	0.219	0.641	0.008	0.927
BMI	*SOD2*	**20.032**	** < 0.001**	0.353	0.553	0.055	0.815
	*NOS2*	**60.488**	** < 0.001**	1.392	0.239	0.156	0.693
Smoking	*SOD2*	**26.653**	** < 0.001**	0.136	0.712	0.018	0.894
	***NOS2***	**70.277**	** < 0.001**	**4.114**	**0.044**	**4.401**	**0.037**

*p < 0.05 are in bold.

## Discussion

BC is the second most common cancer of the genitourinary system, and its incidence is steadily rising worldwide, especially in developed countries^43^. Moreover, BC is a severe health problem of the genitourinary system and is characterised by a high risk of recurrence. Recurrent BC has a critical impact on survival time because the recurrent form of this cancer is more aggressive in its growth pattern than the original lesion^44^. Despite numerous studies, the full mechanism of BC development remains unknown. Nevertheless, a growing body of evidence suggests a crucial role of oxidative stress in the development of BC^14^. Although oxidative stress is a physiological process, its excessive activation may contribute to the development of many different human diseases, including inflammatory diseases, neurodegenerative diseases, cardiovascular diseases, diabetes, and cancers. Tumour formation is a multi-step process involving initiation, promotion, and progression, ultimately leading to the clonal expansion of mutated cells. Importantly, previous studies suggest that oxidative stress is a critical mechanism involved in the process of carcinogenesis. The imbalance between the ROS generation and the antioxidant capacity of the cell can lead to oxidative damage to cellular macromolecules (DNA, proteins, lipids), which can cause the formation of mutagenic DNA damage and modulation of intracellular signalling pathways, such as apoptosis, DNA repair mechanisms and cell proliferation^45,46^.

Importantly, BC is a complex polygenic disease caused by major environmental factors and many low-penetrance predisposition genes^47^. Moreover, BC family history is linked with an approximately twofold increased risk, suggesting a common genetic and potential environmental contribution to its aetiology^48,49^. Genome-wide association studies (GWAS), based on a high-density SNP genotyping array, have identified many gene loci associated with BC development. Previously published results of the GWAS studies have revealed several loci related to BC development, including 1p13.3 (*GSTM1*), 2q37.1 (*UGT1A* cluster), 3q28 (*TP63*), 4p16.3 (*TMEM129* and *TACC3-FGFR3*), 5p15.33 (*TERT-CLPTM1L*), 8p22 (*NAT2*), 8q24.21, 8q24.3 (*PSCA*), 18q12.3 (*SLC14A1*), 19q12 (*CCNE1*), 22q13.1 (*CBX6*, *APOBEC3A*) 3q26.2 (*MTNN*), 11p15.5 (*CDKAL1*) and 8q24 (*MYC*)^50–61^.

However, despite numerous data indicating the critical role of oxidative stress in BC pathogenesis, a review of the available literature confirms that only very few available data indicate the relationship of polymorphisms located in genes involved in the production and neutralisation of ROS with modulation of the BC risk^62–65^. Therefore, in the presented study, we assessed the impact of c. 47 C > T (rs4880)—*SOD2*, (c. 1823 C > T (rs2297518) and g.-1026 C > A (rs2779249)—*NOS2* polymorphisms on the BC frequency. In addition, we also reported the analysis of the influence of BC on mRNA expression and the methylation status of the promoter regions of the studied genes.

The first analysed gene in our work is *SOD2* (*MnSOD*), an encoding enzyme that binds to the superoxide byproducts of oxidative phosphorylation and converts them to hydrogen peroxide and diatomic oxygen. Moreover, SOD2 is the only known antioxidant enzyme found in the mitochondria, which are the main site of ROS production during normal cellular metabolism ^66^. Interestingly, *SOD2* is a highly polymorphic gene, so far 40,701 different SNPs have been registered in the public domain of the NCBI dbSNP. However, the c. 47 C > T (rs4880) SNP deserves special attention because it promotes the development of various cancers, including breast, prostate, and lung cancers^67–69^. Previous studies have shown that the T (Val) allele of this SNP contributes to a reduced expression and production of unstable mRNA. Therefore, T carriers were characterised by increased ROS level as compared to C carriers ^24^. Unfortunately, in our work, we did not find any correlation between 47 C > T (rs4880) SNP and the risk of BC development. On the other hand, Nikić et al. (2023) and Hung et al. (2004) found that the risk of urothelial BC was significantly increased among Val allele carriers compared to Ala/Ala homozygote in the Serbian and Italian populations^70,71^. Moreover, this risk was even greater in smokers with at least one variant of the SOD2 Val allele^70^. However, the results of a meta-analysis by Cao et al. (2014) similar to our findings, showed no significant association between the *SOD2* polymorphism and the risk of bladder cancer^72^. These discrepancies result from the limitations of each study related to the analysis of data from single small ethnic groups. Moreover, we found that the patients with BC were characterised by a decreased mRNA expression of *SOD2* compared to controls. However, we did not confirm the significant influence of the Val allele on the decreased *SOD2* expression. We also detected no statistical differences between the *SOD2* expression and TNM staging subgroups/pathomorphological subgroups Nevertheless, our results are consistent with the evidence for a role for SOD2 in cancer development. The reduced expression of *SOD2* may lead to a reduced amount of active enzyme, resulting in decreased superoxide anion neutralisation capacity. Consequently, it contributes to the disruption of cellular redox homeostasis and may cause genetic and/or epigenetic changes leading to dysregulation of oncogenes and tumour suppressor genes that induce carcinogens^73^. On the other hand, a relative decrease in the amount of hydrogen peroxide due to reduced SOD2 activity will deprive the cell of the stimulus initiating apoptosis, thus allowing it to survive and transform into a cancer cell. Previous studies have confirmed that oxidative stress affects signalling pathways related to cell proliferation^74^. Of these, the epidermal growth factor receptor signalling pathway is particularly important, as well as key signalling proteins, such as nuclear erythroid factor 2-related factor 2 (Nrf2), Ras/Raf (proto-oncogenic serine/threonine protein kinase RAF, associated with small GTP-binding protein Ras), the mitogen-activated protein kinases ERK1/2, and MEK, a 3-kinase phosphatidylinositol, phospholipase C, and protein kinase C, all of which are redox-sensitive^75,76^. In addition, ROS change the expression of the *p53* tumour suppressor gene, which is a key factor in apoptosis. Thus, oxidative stress leads to changes in gene expression, cell proliferation, and apoptosis, and plays a significant role in tumour initiation and progression^77,78^. Interestingly, we found that patients with BC showed a reduced methylation level of *SOD2* promoter region. However, we did not note the effect of tumour progression on the status of *SOD2* methylation. Taking into account the reduced level of expression observed in patients, the expected effect of BC development would be an increased level of methylation compared to the control. This phenomenon may result from other forms of epigenetic regulation, including histone modification (such as methylation and acetylation) and nucleosome positioning^79^.

The second gene analysed in our study is *NOS2*, encoding an enzyme involved in the synthesis of NO from L-arginine upon stimulation by pro-inflammatory cytokines. The role of NO in cancer biology remains unclear. NO may play a dual role in tumour progression, as it may act as both a promoter and an anti-cancer factor, depending on its concentration, time of secretion, or cell type^80^. According to the data presented in the public domain of the NCBI dbSNP, there are 17,265 identified SNPs in *NOS2* gene. Among such a large group of polymorphisms, two SNPs deserve special attention. G.-1026 C > A (rs2779249) and 1823 C > T (rs2297518) polymorphisms are associated with the development of various cancers, such as cervical, nasopharyngeal, gastric, lung, prostate carcinoma^81–85^. In the present work, we found a significant link between g.-1026 C > A (rs2779249)—*NOS2* polymorphism and BC occurrence. The nucleotide change from C to A is associated with higher *iNOS* promoter transcriptional activity, thus this polymorphism contributes to increased production of NO^34^. Our case–control studies have shown for the first time that the subjects carrying the C/A genotype were characterised by decreased risk of developing a BC. Interestingly, our additional analyses confirmed that g.-1026 C > A SNP may modulate BC risk in only women populations and non-smoker groups. We found that the C/C genotype increased BC risk in women and non-smokers while this association was not observed in men and smokers. Moreover, heterozygotes of the SNP were characterised by reduced BC risk in only the women population. In the case of the second studied SNP localised in the *NOS2* gene, c.1823 C > T (rs2297518), we did not observe any correlation with BC occurrence. On the contrary, Ryk et al. (2011) showed that T/T homozygotes had a three-fold higher risk of BC in Sweden's population, but once ill, a lower risk for stage progression and a better prognosis^86^. On the other hand, we detected that the heterozygote of c.1823 C > T (rs2297518) SNP was associated with decreased risk of BC development in only the female population. These differences between the male and female populations in oxidative and antioxidant properties may be due to estrogen production in females. Estrogen acts as an antioxidant, scavenging free radicals due to the presence of the phenolic hydroxyl group. Animal studies have shown that post-castration oxidative stress was higher in female rats compared to control females, while no significant difference was observed in post-castration males. On the other hand, estrogen helps increase the production of mitochondrial ROS, which are involved in cell signalling pathways. This discrepancy is because estrogen selectively influences the level of expression of antioxidant enzymes, including *SOD*^87^. In turn, male testosterone may increase intracellular calcium release in cells, leading to an intensification of ROS generation^88–90^. However, in the case of *NOS2* expression in PBMCs, we detected no statistical differences both in the general population and by subgroups of sex, BMI, and smoking. Similarly, in the case of the size of the primary tumour, the status of lymph nodes metastasis and the distant metastasis as well as the grading of histological malignancy, we also observed no differences between studied groups. On the other hand, precious studies showed that BC patients with positive *iNOS* expression in bladder tissue had higher recurrence risks and reduced recurrence-free survival^91,92^. These differences between our and previous studies may be the result of the diversity of the material that was analysed. Our research focuses on the search for potential molecular biomarkers enabling early diagnosis of BC. Therefore, the starting material for our experiments was blood, which is a relatively readily available material. In turn, all previous studies focused on evaluating the expression in cancerous tissue. Moreover, in the case of urine, Swan’s team was not significantly elevated or decreased NOS activity. Therefore, changes in mRNA expression and activity of iNOS are observable only in the cancerous tissue and they are not reflected peripherally, in the blood (in PBMCs). Unfortunately, despite the evidence indicating the association between the polymorphism occurrence and higher *iNOS* promoter transcriptional activity^38^, we also did not observe the influence of both studied SNPs on *NOS2* expression. However, our results confirmed that BC development elevated the methylation status of the *NOS2* promoter region. Consequently, the expected effect of the polymorphism on mRNA expression could be offset by the increased methylation status of the *NOS2* promoter region^93^.

In conclusion, our results indicated that genetic variants in *NOS2* (c.1823C > T; rs2297518 and g.-1026 C > A; rs2779249) may be associated with individual susceptibility to developing BC. In addition, we showed changes in the expression and methylation of the promoters of *SOD2* and *NOS2* in the BC patients without affecting the further progression and metastasis of BC, which confirms the significant influence of oxidative stress in the induction of neoplastic transformation. This knowledge can help identify specific BC molecular markers to facilitate early diagnosis and develop new effective therapeutic strategies. However, we must not forget the limitations of our study. A limitation of our study is primarily a relatively small number of patients, which can be explained by the recruitment at one hospital. In addition, we would like to emphasize that the case–control study presented here is only preliminary and also limited to one ethnic population, which may contribute to the fact that the results cannot be repeated in other populations. Therefore, there is a justified need for further studies in larger patients and other populations, and our results, however very promising, so far should be interpreted with caution and treated as preliminary, intended to set further research directions.

## Materials and Method

### Participants

The study included a total of 230 native, not-related Poles. A group of 116 patients with diagnosed BC hospitalised at the Department of Urology of the Provincial Integrated Hospital in Plock in the years 2021–2022 and 114 volunteers without health problems, were selected randomly without replacement sampling. Participation in the study was voluntary, and all individuals were informed about the purpose and assured of the voluntary nature of the experiment and the confidentiality of their data before expressing written consent in the statement. Finally, participants signed a statement containing consent to participate in the study before starting the experiment. In the case BC patients, the diagnosis was based on the biopsy and histopathological examination based on the 2004 World Health Organization/International Society of Urological Pathology (WHO/ISUP) classification system^40^ and TNM Classification of Malignant Tumours, 8th Edition developed by the Union for International Cancer Control (UICC)^41^. The exclusion criteria for BC patients included: age below 18 previous or current neoplastic diseases other than BC, autoimmune disorders, or the refusal to consent to participate in the study. Also healthy volunteers who did not agree to participate in the study were excluded. Moreover, all qualified participants were interviewed using a structured questionnaire to determine demographic and potential risk factors for BC. Study participants provided information on their age and gender, lifestyle habits, including smoking (categorised as current, former, or never smokers), marital status, profession, diet, body mass index (BMI), co-occurrence diseases (such as hypertension, diabetes and obesity), symptoms, and family history among 1^st^-degree relatives for BC. The validity and reliability of the questionnaires were checked whenever possible. The full study protocol was approved by the Bioethics Committee of the Faculty of Biology and Environmental Protection of the University of Lodz, Poland (approval no. 12/KBBN-UŁ/II/2020-21) and the Bioethics Committee of the Medical University of Lodz (no. RNN/141/21/KE). All procedures were carried out in accordance with guidelines and regulations of the Bioethics Committee of the University of Lodz and the Medical University and the human sample use was in line with the requirements of the Helsinki Declaration. Characteristics of all participants are presented in Table 1.

### Sample collection and DNA/RNA extraction

Four millilitres of venous blood samples were taken from each BC patient and control into BD Vacutainer^®^ EDTA tubes (Becton, Dickinson and Company Sparks, Maryland, USA), coded, and stored at − 20 °C until used. Then, collected blood samples were used for DNA and RNA extraction, using DNA/RNA Extracol Kit (EURX, Gdansk, Poland) as depicted in the manufacturer’s recommendations. Subsequently, DNA concentration and purity were measured by the Bio-Tek Synergy HT Microplate Reader (Bio-Tek Instruments, Winooski, VT, USA), and the results ranged between 10 and 120 ng/μL and 1.8–2.0, respectively. Finally, genomic DNA and total RNA samples were frozen at − 20 °C until further procedures.

### SNP selection and genotyping

The polymorphisms’ selection was made on the basis of analysis of the public domain of the single nucleotide polymorphism database (dbSNP) at the National Center for Biotechnology Information (NCBI, http://www.ncbi.nlm.nih.gov/snp; accessed on 01 December 2022) and the available literature. The criteria for SNPs’ screening contained the minor allele frequency greater than 0.05 in the European population and their localisation in the coding or regulatory region of the genes. Finally, we chose three polymorphisms presented in Table 7.

**Table 7:** Basic information on the *SOD2* and *NOS2* polymorphisms.

Gene	NCBI db SNP ID (rs no)	Position in g.DNA or c.DNA	Base change	Amino Acid change	Chromosome location	Region	Function	MAF* in European population	TaqMan assay ID	Ref
*SOD2*	rs4880	c.47	T > C	p.Val16Ala	6q25.3	Exon	The 16Ala variant with α-helical structure shows normal transportation of the enzyme into the mitochondria, while the 16Val-containing precursor, which has a β-sheet conformation has 30–40% reduced enzymatic activity	C: 0.498	C_8709053_10	^94^
*NOS2* (*iNOS*)	rs2297518	c.1823	C > T	p.Ser608Leu	17q11.2	Exon	This substitution contributes to an increase in iNOS activity	T: 0.198	C_11889257_10	^34^
	rs2779249	g.-1026	C > A	–	17q11.2	Exon	The nucleotide change from C to A is associated with higher iNOS promoter transcriptional activity	A:0.295	C_2593689_10	^34^

*Minor allele frequency (MAF) in European population.

The genotype profiling was performed using the TaqMan™ SNP genotyping technology (Thermo Fischer Scientific, CA, USA). The real-time polymerase chain reactions (real-time PCR) were performed in a CFX96™ Real-Time PCR Detection System Thermal Cycler (Bio-Rad Laboratories, Inc., Hercules, CA, USA), using specific TaqMan™ probes (assay IDs: C_8709053_10; C_11889257_10; C___2593689_10) and RT PCR Mix Probe (A&A Biotechnology, Gdynia, Poland) by the manufacturer’s instruction (details of thermal cycling conditions for amplifying PCR products are presented in Table 8).

**Table 8:** Thermal profile of real-time PCR used in this study.

Real-time PCR conditions			
Step	AmpliTaq Gold Enzyme Activation	Denature	Anneal
Temperature	95 °C	95 °C	60 °C
Time	3 min	30 s	60 s
Number of cycles	1	40	

### cDNA synthesis and mRNA expression levels

The High-Capacity cDNA Reverse Transcription Kit (Applied Biosystems, Foster City, CA, USA) was used for the reverse transcription of total RNA to cDNA according to the manufacturer’s protocols. Briefly, reverse transcriptase substrates included MultiScribe^®^ Reverse Transcriptase, 10 × RT random primers, 25 × dNTP Mix (100 mM), nuclease-free water, 10 × RT buffer, and total RNA (0.5 ng/μL). The total reaction volume was 20 μL. The reaction was performed using a C1000™ programmed thermal cycler (Bio-Rad Laboratories, Inc., Hercules, CA, USA), and the thermal profile of the reverse transcriptase PCR was as follows: 10 min at 25 °C (enzyme activation), 37 °C for 120 min (proper synthesis of cDNA), and 85 °C for 5 min (enzyme inactivation). Then, mRNA expression was determined by real-time PCR using species-specific TaqMan Gene Expression Assay (*SOD2*—assay ID Hs00167309_m1, *NOS2* assay ID Hs01075529_m1 and *18S* as housekeeping gene—assay ID Hs99999901_s1; Thermo Fisher Scientific, Waltham, MA, USA) and RT PCR Mix Probe (A&A Biotechnology, Gdynia, Poland) by the manufacturer’s protocol (presented in Table 8) on CFX96™ Real-Time PCR Detection System Thermal Cycler (Bio-Rad Laboratories, Inc., Hercules, CA, USA). The real-time PCR mixture consisted of cDNA samples, RT PCR Mix Probe (A&A Biotechnology, Gdynia, Poland), a TaqMan probe (Thermo Fisher Scientific, Waltham, MA, USA), and RNAse-free water. Finally, the relative mRNA expression level was calculated as the 2^-ΔCt^ sample, where ΔC_t sample_ = C_t target gene_ − C_t housekeeping gene_^95^.

### Bisulfite treatment and methylation analysis by MS-HRM

The methylation-sensitive high-resolution melting (MS-HRM) was used for the methylation status determination of studied gene promoters^96,97^. For this purpose, in the first step, the gene sequences were checked for the number of promoters and the presence of CpG islands. The EPD eukaryotic promoter database (http://epd.vital-it.ch (accessed December 1, 2022) was used to obtain the promoter sequences of the studied genes^98^. Moreover, the prediction of CpG island occurrence in the promoter regions was made using the EMBOSS Cpgplot bioinformatics tool https://www.ebi.ac.uk/Tools/seqstats/emboss_cpgplot/, Settings: Window: 100, Shift: 1, Obs. /Exp.: 0.6, GC content: 50%). Subsequently, primers were designed using a MethPrimer 2 (http://www.urogene.org/methprimer2/) according to the recommendations provided by Wojdacz et al. (2009)^99^. In the second step, the bisulfite conversion was performed using the CiTi Converter DNA Methylation Kit (A&A Biotechnology, Gdynia, Poland) according to the manufacturer’s instructions. Then, real-time PCR amplification was carried out on the Bio-Rad CFX96 Real-Time PCR Detection System (Bio-Rad Laboratories, Inc., Hercules, CA, USA). The total reaction volume was 10 μL including bisulfite modified DNA template (10 ng/µL), RT PCR Mix EvaGreen^®^ (A&A Biotechnology, Gdynia, Poland), 500 NM of forward and reverse primers, and PCR-grade water. The MS-HRM reaction included steps presented in Table 9. All reactions were performed in duplicate. Moreover, unmethylated and methylated bisulfite-transformed control DNA (CpGenome Human Methylated DNA Standard Set, Merck Millipore Burlington, MA, USA) and CpGenome Human Non-Methylated DNA Standard Set, Merck Millipore Burlington, MA, USA) were used in varying proportions to maintain accuracy and to control the sensitivity of methylation detection. (0%, 10%, 25%, 50%, 75% and 100% methylated controls). Finally, the Bio-Rad Precision Melt Analysis software (Bio-Rad Laboratories Inc., Hercules, CA, USA) was used to analyse the obtained data.

**Table 9:** Thermal profile of MS-HRM.

Step		Temperature	Duration	Cycles
Enzyme activation		95 °C	3 min	1
Denature		95 °C	30 s	45
Annealing*	*SOD2*	57 °C	60 s	
	*NOS2*	57 °C	60 s	
Extention		72 °C	60 s	
HRM		95–60 °C	0.2 s for 1 degree	1

*At optimal primer temperatures.

### Statistical analysis

Statistical analyses were performed with Statistica 12 (Statsoft, Tulsa, OK, USA) and SigmaPlot 11.0 (Systat Software Inc., San Jose, CA, USA). For each polymorphism, χ2 test was used for the assessment of the Hardy–Weinberg equilibrium (HWE) to compare the observed and expected genotype frequencies. The association between case–control status and each studied SNP, measured by the odds ratio (OR) and its corresponding 95% confidence interval (CI), was evaluated by an unconditional multiple logistic regression model, both with and without adjustment for sex. The association between the combined genotypes of the *SOD2* and *NOS2* SNPs and the risk of this disease was also evaluated in the same way as single SNPs. In addition, we also evaluated the potential SNP-SNP interactions according to Mario Cortina-Borja et al.’ (2009) recommendations^35^. Linkage disequilibrium (LD) and haplotype distribution analysis was assessed using the SHEsisPlus software (http://shesisplus.bio-x.cn/SHEsis.html, accessed on 23 December 2022)^36–39^. In the same way as single polymorphism, we evaluated the association between the BC patients and controls for each SNP in the male/female population or non-smoker/smoker groups or subpopulations with the normal body weight/overweight/obesity group. The frequency distributions of various clinical characteristics for the different genotypes of each polymorphism were assessed by Pearson’s χ2 test. Data of mRNA expression and demographics and baseline characteristics of patients were analysed by the Mann–Whitney test or non-normally distributed data or Student’s t-test for normally distributed data. To assess the *SOD2* and *NOS2* gene expression levels between respective genotypes of the analysed SNPs, the Kruskal–Wallis One Way Analysis of Variance on Ranks was applied. Moreover, the two-way ANOVA analyses were used to the evaluation of effects of gender/BMI/cigarette smoking and BC on mRNA expression. Finally, the Bonferroni test was used as a post hoc test. The values of *p* < 0.05 were considered statistically significant.

### Supplementary Information

Supplementary Information.

## Data Availability

The data that support the findings of this study are available on http://hdl.handle.net/11089/46226.

## References

[1] Andersson KE, Arner A (2004). Urinary bladder contraction and relaxation: Physiology and pathophysiology. Physiol. Rev..

[2] Mushtaq J, Thurairaja R, Nair R (2019). Bladder cancer. Surgery..

[3] Mostafa MH, Sheweita S, O’Connor PJ (1999). Relationship between schistosomiasis and bladder cancer. Clin. Microbiol. Rev..

[4] Bray F, Ferlay J, Soerjomataram I, Siegel RL, Torre LA, Jemal A (2018). Global cancer statistics 2018: GLOBOCAN estimates of incidence and mortality worldwide for 36 cancers in 185 countries. CA Cancer J. Clin..

[5] Global Cancer Observatory (2021). GLOBOCAN 2020 v10: Cancer incidence and mortality worldwide.

[6] Siegel RL, Miller KD, Jemal A (2019). Cancer statistics, 2019. CA Cancer J. Clin..

[7] García-Pérez J, Pollán M, Boldo E, Pérez-Gómez B, Aragonés N, Lope V, Ramis R, Vidal E, López-Abente G (2009). Mortality due to lung, laryngeal and bladder cancer in towns lying in the vicinity of combustion installations. Sci. Total Environ..

[8] Peralta-Videa JR, Lopez ML, Narayan M, Saupe G, Gardea-Torresdey J (2009). The biochemistry of environmental heavy metal uptake by plants: Implications for the food chain. Int. J. Biochem. Cell Biol..

[9] Snyderwine EG, Sinha R, Felton JS, Ferguson LR (2002). Highlights of the eighth international conference on carcinogen-ic/mutagenic N-substituted aryl compounds. Mutat. Res..

[10] Sun JW, Zhao LG, Yang Y, Ma X, Wang YY, Xiang YB (2015). Obesity and risk of bladder cancer: A dose-response meta-analysis of 15 cohort studies. PLoS ONE.

[11] Michaud DS, Kogevinas M, Cantor KP, Villanueva CM, Garcia-Closas M, Rothman N, Malats N, Real FX, Serra C, García-Closas R (2007). Total fluid and water consumption and the joint effect of exposure to disinfection by-products on risk of bladder cancer. Environ. Health Perspect..

[12] Michaud DS, Spiegelman D, Clinton SK, Rimm EB, Curhan GC, Willett WC, Giovannucci EL (1999). Fluid intake and the risk of bladder cancer in men. N. Engl. J. Med..

[13] Gu J, Wu X (2011). Genetic susceptibility to bladder cancer risk and outcome. Pers. Med..

[14] Wigner P, Grębowski R, Bijak M, Saluk-Bijak J, Szemraj J (2021). The Interplay between oxidative stress, inflammation and angiogenesis in bladder cancer development. Int. J. Mol. Sci..

[15] Jeon SH, Park JH, Chang SG (2007). Expression of antioxidant enzymes (catalase, superoxide dismutase, and glutathione peroxi-dase) in human bladder cancer. Korean J. Urol..

[16] Hung RJ, Boffetta P, Brennan P, Malaveille C, Gelatti U, Placidi D, Carta A, Hautefeuille A, Porru S (2004). Genetic polymorphisms of MPO, COMT, MnSOD, NQO1, interactions with environmental exposures and bladder cancer risk. Carcinogen.

[17] Lepara Z, Lepara O, Fajkić A, Rebić D, Alić J, Spahović H (2020). Serum malondialdehyde (MDA) level as a potential biomarker of cancer progression for patients with bladder cancer. Rom. J. Intern. Med..

[18] Szymańska B, Sawicka E, Matuszewski M, Dembowski J, Piwowar A (2020). The dependence between urinary levels of angiogenesis factors, 8-Iso-prostaglandin F2α, ɣ-Synuclein, and Interleukin-13 in patients with bladder cancer: A pilot study. J. Oncol..

[19] Kiliç S, Bayraktar N, Beytur A, Ergin H, Bayraktar M, Eǧri M, Egri M (2006). Can the levels of nitric oxide in the urine, serum and tumor tissue be putative markers for bladder cancer?. Int. J. Urol..

[20] Gecit I, Aslan M, Gunes M, Pirincci N, Esen R, Demir H, Ceylan K (2012). Serum prolidase activity, oxidative stress, and nitric ox-ide levels in patients with bladder cancer. J. Cancer Res. Clin. Oncol..

[21] Sharifi-Rad M (2020). Lifestyle, oxidative stress, and antioxidants: Back and forth in the pathophysiology of chronic diseases. Front Physiol..

[22] Yuzhalin AE, Kutikhin AG (2012). Inherited variations in the SOD and GPX gene families and cancer risk. Free Radic. Res..

[23] Cormio A, Sanguedolce F, Musicco C, Pesce V, Calò G, Bufo P, Carrieri G, Cormio L (2017). Mitochondrial dysfunctions in bladder cancer: Exploring their role as disease markers and potential therapeutic targets. Crit Rev Oncol Hematol..

[24] Sutton A, Imbert A, Igoudjil A, Descatoire V, Cazanave S, Pessayre D, Degoul F (2005). The manganese superoxide dismutase Ala16Val dimorphism modulates both mitochondrial import and mRNA stability. Pharmacogenet. Genom..

[25] Schieber M, Chandel NS (2014). ROS function in redox signaling and oxidative stress. Curr Biol..

[26] Marnett LJ, Riggins JN, West JD (2003). Endogenous generation of reactive oxidants and electrophiles and their reactions with DNA and protein. J. Clin. Investig..

[27] Park SG, Kim SH, Kim KY, Yu SN, Choi HD, Kim YW, Nam HW, Seo YK, Ahn SC (2017). Toyocamycin induces apoptosis via the crosstalk between reactive oxygen species and p38/ERK MAPKs signaling pathway in human prostate cancer PC-3 cells. Pharmacol. Rep..

[28] Kao SJ, Lee WJ, Chang JH, Chow JM, Chung CL, Hung WY, Chien MH (2017). Suppression of reactive oxygen species-mediated ERK and JNK activation sensitizes dihydromyricetin-induced mitochondrial apoptosis in human non-small cell lung can-cer. Environ. Toxicol..

[29] Furlan D, Trapani D, Berrino E, Debernardi C, Panero M, Libera L, Sahnane N, Riva C, Tibiletti MG, Sessa F (2017). Oxidative DNA damage induces hypomethylation in a compromised base excision repair colorectal tumourigenesis. Br. J. Cancer.

[30] Milkovic L, Siems W, Siems R, Zarkovic N (2014). Oxidative stress and antioxidants in carcinogenesis and integrative therapy of cancer. Curr. Pharm. Des..

[31] The Cancer Genome Atlas Research Network (2014). Comprehensive molecular characterization of urothelial bladder carcinoma. Nature.

[32] Vannini F, Kashfi K, Nath N (2015). The dual role of iNOS in cancer. Redox Biol..

[33] Lin Z, Chen S, Ye C, Zhu S (2003). Nitric oxide synthase expression in human bladder cancer and its relation to angiogenesis. Urol. Res..

[34] Dhillon SS, Mastropaolo LA, Murchie R, Griffiths C, Thöni C, Elkadri A, Xu W, Mack A, Walters T, Guo C, Mack D, Huynh H, Baksh S, Silverberg MS, Brumell JH, Snapper SB, Muise AM (2014). Higher activity of the inducible nitric oxide synthase contributes to very early onset inflammatory bowel disease. Clin Transl Gastroenterol..

[35] Cortina-Borja M, Smith AD, Combarros O, Lehmann DJ (2009). The synergy factor: A statistic to measure interactions in complex diseases. BMC Res. Notes.

[36] Qin ZS, Niu T, Liu JS (2002). Partition-ligation-expectation-maximization algorithm for haplotype inference with single-nucleotide polymorphisms. Am. J. Hum. Genet..

[37] Shen J, Li Z, Chen J, Song Z, Zhou Z, Shi Y (2026). SHEsisPlus, a toolset for genetic studies on polyploid species. Sci. Rep..

[38] Shi YY, He L (2005). SHEsis, a powerful software platform for analyses of linkage disequilibrium, haplotype construction, and genetic association at polymorphism loci. Cell Res..

[39] Li Z, Zhang Z, He Z, Tang W, Li T, Zeng Z, He L, Shi Y (2009). A partition-ligation-combination-subdivision EM algorithm for haplotype inference with multiallelic markers. Cell Res..

[40] Brierley JD, Gospodarowicz MK, Wittekind C (2017). TNM classification of malignant tumours.

[41] Miyamoto H, Miller JS, Fajardo DA, Lee TK, Netto GJ, Epstein JI (2010). Non-invasive papillary urothelial neoplasms: the 2004 WHO/ISUP classification system. Pathol Int..

[42] Shastry BS (2009). SNPs: impact on gene function and phenotype. Methods Mol. Biol..

[43] Saginala K, Barsouk A, Aluru JS, Rawla P, Padala SA, Barsouk A (2020). Epidemiology of bladder cancer. Med. Sci..

[44] Gogalic S, Sauer U, Doppler S, Preininger C (2015). Bladder cancer biomarker array to detect aberrant levels of proteins in urine. Analyst.

[45] Visconti R, Grieco D (2009). New insights on oxidative stress in cancer. Curr. Opin. Drug. Discov. Devel..

[46] Havermann S, Büchter C, Koch K, Wätjen W, Roberts S, Kehrer J, Klotz LO (2015). Role of oxidative stress in the process of carcinogenesis. Studies on experimental toxicology and pharmacology. Oxidative stress in applied basic research and clinical practice.

[47] Gu J, Wu X (2011). Genetic susceptibility to bladder cancer risk and outcome. Pers. Med..

[48] Kantor AF, Hartge P, Hoover RN, Fraumeni JF (1985). Familial and environmental interactions in bladder cancer risk. Int. J. Cancer..

[49] Murta-Nascimento C, Silverman DT, Kogevinas M, Garcia-Closas M, Rothman N, Tardon A, Garcia-Closas R, Serra C, Carrato A, Villanueva C, Dosemeci M, Real FX, Malats N (2007). Risk of bladder cancer associated with family history of cancer: do low-penetrance polymorphisms account for the increase in risk?. Cancer Epidemiol. Biomarkers Prev..

[50] García-Closas M (2005). NAT2 slow acetylation, GSTM1 null genotype, and risk of bladder cancer: results from the Spanish bladder cancer study and meta-analyses. Lancet.

[51] Garcia-Closas M, Ye Y, Rothman N, Figueroa JD, Malats N, Dinney CP, Chatterjee N, Prokunina-Olsson L, Wang Z, Lin J (2011). A genome-wide association study of bladder cancer identifies a new susceptibility locus within SLC14A1, a urea transporter gene on chromosome 18q12.3. Hum. Mol. Genet..

[52] Kiemeney LA, Thorlacius S, Sulem P, Geller F, Aben KK, Stacey SN, Gudmundsson J, Jakobsdottir M, Bergthorsson JT, Sigurdsson A (2008). Sequence variant on 8q24 confers susceptibility to urinary bladder cancer. Nat. Genet..

[53] Moore LE, Baris DR, Figueroa JD, Garcia-Closas M, Karagas MR, Schwenn MR, Johnson AT, Lubin JH, Hein DW, Dagnall CL (2011). GSTM1 null and NAT2 slow acetylation genotypes, smoking intensity and bladder cancer risk: results from the New England bladder cancer study and NAT2 meta-analysis. Carcinogenesis.

[54] Rothman N, Garcia-Closas M, Chatterjee N, Malats N, Wu X, Figueroa JD, Real FX, Van Den Berg D, Matullo G, Baris D (2010). A multi-stage genome-wide association study of bladder cancer identifies multiple susceptibility loci. Nat. Genet..

[55] Wu X, Ye Y, Kiemeney LA, Sulem P, Rafnar T, Matullo G, Seminara D, Yoshida T, Saeki N, Andrew AS (2009). Genetic variation in the prostate stem cell antigen gene PSCA confers susceptibility to urinary bladder cancer. Nat. Genet..

[56] Kiemeney LA, Sulem P, Besenbacher S, Vermeulen SH, Sigurdsson A, Thorleifsson G, Gudbjartsson DF, Stacey SN, Gudmundsson J, Zanon C (2010). A sequence variant at 4p16.3 confers susceptibility to urinary bladder cancer. Nat. Genet..

[57] Rafnar T, Vermeulen SH, Sulem P (2011). European genome-wide association study identifies SLC14A1 as a new urinary bladder cancer susceptibility gene. Hum. Mol. Genet..

[58] Tang W, Fu YP, Figueroa JD, Malats N, Garcia-Closas M, Chatterjee N, Kogevinas M, Baris D, Thun M, Hall JL (2012). Mapping of the UGT1A locus identifies an uncommon coding variant that affects mRNA expression and protects from bladder cancer. Hum. Mol. Genet..

[59] Rafnar T, Sulem P, Stacey SN, Geller F, Gudmundsson J, Sigurdsson A, Jakobsdottir M, Helgadottir H, Thorlacius S, Aben KK (2009). Sequence variants at the TERT-CLPTM1L locus associate with many cancer types. Nat. Genet..

[60] Figueroa JD, Ye Y, Siddiq A (2014). Genome-wide association study identifies multiple loci associated with bladder cancer risk. Human Mol. Genet..

[61] Kiemeney LA, Grotenhuis AJ, Vermeulen SH, Wu X (2009). Genome-wide association studies in bladder cancer: first results and potential relevance. Curr. Opin. Urol..

[62] Amasyali AS, Kucukgergin C, Erdem S, Sanli O, Seckin S, Nane I (2012). Nitric oxide synthase (eNOS4a/b) gene polymorphism is associated with tumor recurrence and progression in superficial bladder cancer cases. J. Urol..

[63] Savic-Radojevic A, Djukic T, Simic T, Pljesa-Ercegovac M, Dragicevic D, Pekmezovic T (2013). GSTM1-null and GSTA1-low activity genotypes are associated with enhanced oxidative damage in bladder cancer. Redox Rep..

[64] Park SJ, Zhao H, Spitz MR, Grossman HB, Wu X (2003). An association between NQO1 genetic polymorphism and risk of bladder cancer. Mutat. Res. Genet. Toxicol. Environ. Mutagen..

[65] Wei H, Kamat A, Chen M, Ke HL, Chang DW, Yin J (2012). Association of polymorphisms in oxidative stress genes with clinical outcomes for bladder cancer treated with Bacillus Calmette-Guerin. PLoS ONE.

[66] Andreyev AY, Kushnareva YE, Starkov AA (2005). Mitochondrial metabolism of reactive oxygen species. Biochemistry.

[67] Crawford A, Fassett RG, Geraghty DP, Kunde DA, Ball MJ, Robertson IK, Coombes JS (2012). Relationships between single nucleotide polymorphisms of antioxidant enzymes and disease. Gene.

[68] Blein S, Berndt S, Joshi AD, Campa D, Ziegler RG, Riboli E, Cox DG, Gaudet MM, Stevens VL, Diver WR (2014). Factors associated with oxidative stress and cancer risk in the breast and prostate cancer cohort consortium. Free Radic. Res..

[69] Jabir FA, Hoidy WH (2018). Pharmacogenetics as personalized medicine: Association investigation of SOD2 rs4880, CYP2C19 rs4244285, and FCGR2A rs1801274 polymorphisms in a breast cancer population in Iraqi women. Clin. Breast Cancer.

[70] Nikic P, Dragicevic D, Jerotic D, Savic S, Djukic T, Stankovic B, Kovacevic L, Simic T, Matic M (2023). Polymorphisms of antioxidant enzymes SOD2 (rs4880) and GPX1 (rs1050450) are associated with bladder cancer risk or its aggressiveness. Medicina.

[71] Hung RJ, Boffetta P, Brennan P, Malaveille C, Gelatti U, Placidi D, Carta A, Hautefeuille A, Porru S (2004). Genetic polymorphisms of MPO, COMT, MnSOD, NQO1, interactions with environmental exposures and bladder cancer risk. Carcinogenesis.

[72] Cao M, Mu X, Jiang C, Yang G, Chen H, Xue W (2014). Single-nucleotide polymorphisms of GPX1 and MnSOD and susceptibility to bladder cancer: A systematic review and meta-analysis. Tumor Biol..

[73] Murata M, Thanan R, Ma N, Kawanishi S (2012). Role of nitrative and oxidative DNA damage in inflammation-related carcinogenesis. J. Biomed. Biotechnol..

[74] Klaunig JE, Kamendulis LM, Hocevar BA (2010). Oxidative stress and oxidative damage in carcinogenesis. Toxicol. Pathol..

[75] Huo L, Li CW, Huang TH, Lam YC, Xia W, Tu C, Chang WC, Hsu JL, Lee DF, Nie L (2014). Activation of Keap1/Nrf2 signaling pathway by nuclear epidermal growth factor receptor in cancer cells. Am. J. Transl. Res..

[76] Korbecki J, Baranowska-Bosiacka I, Gutowska I, Chlubek D (2013). The effect of reactive oxygen species on the synthesis of prostanoids from arachidonic acid. J. Physiol. Pharmacol..

[77] Matsuzawa A, Ichijo H (2008). Redox control of cell fate by MAP kinase: Physiological roles of ASK1-MAP kinase pathway in stress signaling. Biochim. Biophys. Acta Gen. Subj..

[78] Barrera G (2012). Oxidative stress and lipid peroxidation products in cancer progression and therapy. ISRN Oncol..

[79] Portela A, Esteller M (2010). Epigenetic modifications and human disease. Nat Biotechnol..

[80] Jenkins DC, Charles IG, Thomsen LL, Moss DW, Holmes LS, Baylis SA, Rhodes P, Westmore K, Emson PC, Moncada S (1995). Roles of nitric oxide in tumor growth. Proc. Natl. Acad. Sci. USA.

[81] Yamamoto Y, Kiyohara C, Suetsugu-Ogata S, Hamada N, Nakanishi Y (2017). Biological interaction of cigarette smoking on the association between genetic polymorphisms involved in inflammation and the risk of lung cancer: A case-control study in Japan. Oncol. Lett..

[82] Zhu Y, Jiang H, Chen Z, Lu B, Li J, Peng Y, Shen X (2018). The genetic association between iNOS and eNOS polymorphisms and gastric cancer risk: a meta-analysis. Onco. Targets. Ther..

[83] Lee KM, Kang D, Park SK, Berndt SI, Reding D, Chatterjee N, Chanock S, Huang WY, Hayes RB (2009). Nitric oxide synthase gene polymorphisms and prostate cancer risk. Carcinogenesis.

[84] Li P, Meng J, Zhai Y, Zhang H, Yu L, Wang Z, Zhang X, Cao P, Chen X, Han Y, Zhang Y, Chen H, Ling Y, Li Y, Cui Y, Bei JX, Zeng YX, He F, Zhou G (2015). Argonaute 2 and nasopharyngeal carcinoma: a genetic association study and functional analysis. BMC Cancer.

[85] Sowjanya AP, Rao M, Vedantham H, Kalpana B, Poli UR, Marks MA, Sujatha M (2016). Correlation of plasma nitrite/nitrate levels and inducible nitric oxide gene expression among women with cervical abnormalities and cancer. Nitric Oxide Biol. Chem..

[86] Ryk C, Wiklund NP, Nyberg T, De Verdier PJ (2011). Ser608Leu polymorphisms in the nitric oxide synthase-2 gene may influence urinary bladder cancer pathogenesis. Scand. J. Urol. Nephrol..

[87] Kander MC, Cui Y, Liu Z (2017). Gender difference in oxidative stress: A new look at the mechanisms for cardiovascular diseases. J. Cell. Mol. Med..

[88] Vicencio JM, Ibarra C, Estrada M, Chiong M, Soto D, Parra V, Diaz-Araya G, Jaimovich E, Lavandero S (2006). Testosterone induces an intracellular calcium increase by a nongenomic mechanism in cultured rat cardiac myocytes. Endocrinology.

[89] Tenkorang MA, Snyder B, Cunningham RL (2018). Sex-related differences in oxidative stress and neurodegeneration. Steroids.

[90] Holmes S, Singh M, Su C, Cunningham RL (2016). Effects of oxidative stress and testosterone on pro-inflammatory signaling in a female rat dopaminergic neuronal cell line. Endocrinology.

[91] Sandes EO, Faletti AG, Riveros MD, Vidal M (2005). Expression of inducible nitric oxide synthase in tumoral and non-tumoral epithelia from bladder cancer patients. Nitric Oxide.

[92] Chih-Ming Lu, Chiu AW, Huang Y-L, Lee Y-H, Ko Y-C (2008). Association between positive iNOS mRNA expression and recurrence-free survival among patients with non-muscle-invasive bladder cancer. Tzu Chi Med. J..

[93] Johnson AD, Zhang Y, Papp AC, Pinsonneault JK, Lim E, Saffen D, Dai Z, Wang D, Sadée W (2008). Polymorphisms affecting gene transcription and mRNA processing in pharmacogenetic candidate genes: Detection through allelic expression imbalance in human target tissues. Pharmacogenet. Genom..

[94] Sutton A, Khoury MH, Prip-Buus C, Cepanec C, Pessayre D, Degoul F (2003). The Ala16Val genetic dimorphism modulates the import of human manganese superoxide dismutase into rat liver mitochondria. Pharmacogenetics.

[95] Rao X, Huang X, Zhou Z, Lin X (2013). An improvement of the 2ˆ(-delta delta CT) method for quantitative real-time polymerase chain reaction data analysis. Biostat. Bioinforma. Biomath..

[96] Wojdacz TK, Dobrovic A, Hansen LL (1903). Methylation-sensitive high-resolution melting. Nat. Protoc..

[97] Wojdacz TK, Dobrovic A (2007). Methylation-sensitive high resolution melting (MS-HRM): A new approach for sensitive and high-throughput assessment of methylation. Nucleic Acids Res..

[98] Dreos R, Ambrosini G, Groux R, Périer RC, Bucher P (2017). The eukaryotic promoter database in its 30^th^ year: Focus on non-vertebrate organisms. Nucleic Acids Res..

[99] Wojdacz TK, Borgbo T, Hansen LL (2009). Primer design versus PCR bias in methylation independent PCR amplifications. Epigenetics.

