# Supplementary material for: The role of SOD2 and NOS2 genes in the molecular aspect of bladder cancer pathophysiology
Source: Sci Rep. 2023 Sep 2;13:14491. doi: 10.1038/s41598-023-41752-8 (PMC10475080; doi:10.1038/s41598-023-41752-8)
Supplement: Supplementary file 1 — Supplementary Information. [file 41598_2023_41752_MOESM1_ESM.docx]

**Supplementary Table 1.** Gene-gene interactions of rs4880 (*SOD2*), rs2297518 (*NOS2*), and rs2779249 (*NOS2*) and the risk of BC occurrence.

| Combined genotype | Control (n = 114) | | BC (n = 116) | | Crude OR (95% CI)* | *p* | Adjusted OR (95% CI)* | *p* |
| --- | --- | --- | --- | --- | --- | --- | --- | --- |
|  | Number | Frequency | Number | Frequency |  |  |  |  |
| **c. 47C>T – *SOD2* (rs4880) and c.1823 C>T (p. Ser608Leu) – *NOS2* (rs2297518)** | | | | | | | | |
| T/T-T/T | 1 | 0.009 | 1 | 0.009 | 0.983 (0.061-15.901) | 0.990 | 1.226 (0.074-20.403) | 0.887 |
| T/T-T/C | 9 | 0.079 | 5 | 0.043 | 0.526 (0.171-1.619) | 0.262 | 0.532 (0.172-1.644) | 0.273 |
| T/T-C/C | 19 | 0.167 | 15 | 0.129 | 0.743 (0.357-1.545) | 0.426 | 0.761 (0.365-1.588) | 0.467 |
| T/C-T/T | 1 | 0.009 | 0 | 0.000 | 0.000 (0.000-+inf.) | 0.991 | 0.000 (0.000-+inf.) | 0.991 |
| T/C-T/C | 18 | 0.158 | 19 | 0.164 | 1.045 (0.517-2.112) | 0.903 | 1.040 (0.514-2.107) | 0.912 |
| T/C-C/C | 37 | 0.325 | 47 | 0.405 | 1.418 (0.826-2.431) | 0.205 | 1.383 (0.804-2.380) | 0.241 |
| C/C-T/T | 1 | 0.009 | 1 | 0.009 | 0.983 (0.061-15.901) | 0.990 | 0.892 (0.055-14.508) | 0.936 |
| C/C-T/C | 8 | 0.070 | 8 | 0.069 | 0.981 (0.355-2.711) | 0.971 | 1.003 (0.362-2.782) | 0.995 |
| C/C-C/C | 20 | 0.175 | 20 | 0.172 | 0.979 (0.495-1.936) | 0.952 | 0.985 (0.49701.952) | 0.966 |
| **c. 47C>T – *SOD2* (rs4880) and g.-1026 C>A – *NOS2* (rs2779249)** | | | | | | | | |
| T/T-C/C | 16 | 0.140 | 14 | 0.121 | 0.841 (0.390-1.814) | 0.658 | 0.837 (0.387-1.810) | 0.652 |
| T/T-C/A | 11 | 0.096 | 4 | 0.034 | 0.334 (0.103-1.082) | 0.068 | 0.358 (0.109-1.175) | 0.090 |
| T/T-A/A | 2 | 0.018 | 3 | 0.026 | 1.487 (0.244-9.068) | 0.667 | 1.535 (0.250-9.421) | 0.643 |
| T/C-C/C | 27 | 0.237 | 35 | 0.302 | 1.392 (0.775-2.502) | 0.268 | 1.343 (0.743-2.426) | 0.329 |
| T/C-C/A | 25 | 0.219 | 23 | 0.198 | 0.880 (0.466-1.664) | 0.695 | 0.888 (0.469-1.682) | 0.716 |
| T/C-A/A | 4 | 0.035 | 8 | 0.069 | 2.307 (0.596-6.964) | 0.257 | 2.005 (0.585-6.874) | 0.268 |
| C/C-C/C | 14 | 0.123 | 16 | 0.138 | 1.143 (0.530-2.466) | 0.734 | 1.154 (0.534-2.495) | 0.716 |
| C/C-C/A | 15 | 0.132 | 10 | 0.086 | 0.623 (0.267-1.451) | 0.272 | 0.624 (0.267-1.456) | 0.275 |
| C/C-A/A | 0 | 0.000 | 3 | 0.026 | 4039797.021 (0.000-+inf.) | 0.989 | 4098866.901 (0.000-+inf.) | 0.989 |

* crude OR means OR calculated with conventional logistic regression; for the significant outcomes, adjusted OR means OR calculated with conventional logistic regression adjusted sex

**Supplementary Table 2.** Synergy factor analysis.

| **Genes** | **Polymorphism** | **Subjects** | **Synergy factor ^a^** | ***p-*value ^b^** | **Type interaction ^a^** |
| --- | --- | --- | --- | --- | --- |
| *SOD2 × NOS2* | c.47 C>T (p.Val16Ala) (rs4880) – *SOD2* × c.1823 C>T (p. Ser608Leu) (rs2297518) – *NOS2* | T carriers – T carriers | 0.779 | 0.703 | Synergistic |
| *SOD2 × NOS2* | c.47 C>T (p.Val16Ala) (rs4880) – *SOD2* × g.-1026 C>A (rs2779249) – *NOS2* | T carriers – A carriers | 1.047 | 0.940 | Antagonistic |

^a^ All SF relate to the risk of BC. The cited genotypes were treated as risk factors unless otherwise stated; the terms, ‘risk’ and ‘protective’ factors, refer to associations, i.e. no causality is implied. Note that synergy (antagonism) between risk factors will produce a SF > 1 (< 1), while synergy (antagonism) between protective factors will give a SF < 1 (> 1).

^b^ All *p*-values are before correction for multiple testing, whether or not relevant.

**Supplementary Table 3.** Distribution of haplotypes of the studied polymorphisms of the *NOS2* and BC risk.

| Haplotype | Control (n = 114) | | BC (n = 116) | | Crude ORs (95% CIs)* | *p* |
| --- | --- | --- | --- | --- | --- | --- |
|  | Number | Frequency | Number | Frequency |  |  |
| **c.1823 C>T (p. Ser608Leu) – *NOS2* (rs2297518) and g.-1026 C>A – *NOS2* (rs2779249)** | | | | | | |
| TA | 30 | 0.131 | 28 | 0.120 | 0.905 (0.522-1.571) | 0.725 |
| CC | 154 | 0.675 | 159 | 0.685 | 1.046 (0.707-1.548) | 0.819 |
| CA | 33 | 0.144 | 37 | 0.159 | 1.121 (0.673-1.866) | 0.659 |
| TC | 11 | 0.048 | 8 | 0.034 | 0.704 (0.278-1.785) | 0.458 |

* crude OR means OR calculated with conventional logistic regression; for the significant outcomes

**Supplementary Table 4.** Correlations between studied SNPs and the clinical characteristics of patients with BC.

| **c. 47 C>T – *SOD2* (rs4880)** | | | | | | |
| --- | --- | --- | --- | --- | --- | --- |
| **Characteristics** | **Status** | **Genotype**  **Frequency** | | | **χ2** | ***p*** |
|  |  | **CC** | **CT** | **TT** |  |  |
| **Primary tumour size** | Ta | 0.346 | 0.420 | 0.471 | 1.887 | 0.756 |
|  | T1 | 0.500 | 0.400 | 0.294 |  |  |
|  | ≥T2 | 0.154 | 0.180 | 0.235 |  |  |
| **Lymph node metastasis** | N0 | 0.846 | 0.820 | 0.765 | 0.462 | 0.794 |
|  | ≥N1 | 0.154 | 0.180 | 0.235 |  |  |
| **Distant metastasis** | M0 | 0.846 | 0.820 | 0.765 | 0.462 | 0.794 |
|  | M1 | 0.154 | 0.180 | 0.235 |  |  |
| **Pathomorphology of nonmuscle-invasive tumors** | papillary urothelial neoplasm of low malignant potential (PUN-LMP) | 0.111 | 0.172 | 0.389 | 5.863 | 0.210 |
|  | low-grade papillary urothelial carcinoma | 0.444 | 0.431 | 0.278 |  |  |
|  | high-grade papillary urothelial carcinoma | 0.444 | 0.397 | 0.333 |  |  |
| **c.1823 C>T (p. Ser608Leu) – *NOS2* (rs2297518)** | | | | | | |
| **Characteristics** | **Status** | **Genotype**  **Frequency** | | | **χ2** | ***p*** |
|  |  | **GG** | **GA** | **AA** |  |  |
| **Primary tumour size** | Ta | 0.406 | 0.370 | 1.000 | 3.087 | 0.543 |
|  | T1 | 0.406 | 0.44 | 0.000 |  |  |
|  | ≥T2 | 0.188 | 0.185 | 0.000 |  |  |
| **Lymph node metastasis** | N0 | 0.813 | 0.815 | 1.000 | 0.458 | 0.795 |
|  | ≥N1 | 0.187 | 0.185 | 0.000 |  |  |
| **Distant metastasis** | M0 | 0.813 | 0.815 | 1.000 | 0.458 | 0.795 |
|  | M1 | 0.187 | 0.185 | 0.000 |  |  |
| **Pathomorphology of nonmuscle-invasive tumors** | papillary urothelial neoplasm of low malignant potential (PUN-LMP) | 0.216 | 0.148 | 0.000 | 5.278 | 0.260 |
|  | low-grade papillary urothelial carcinoma | 0.216 | 0.519 | 1.000 |  |  |
|  | high-grade papillary urothelial carcinoma | 0.432 | 0.333 | 0.000 |  |  |
| **g.-1026 C>A – *NOS2* (rs2779249)** | | | | | | |
| **Characteristics** | **Status** | **Genotype**  **Frequency** | | | **χ2** | ***p*** |
|  |  | **CC** | **CA** | **AA** |  |  |
| **Primary tumour size** | Ta | 0.490 | 0.355 | 0.231 | 3.670 | 0.452 |
|  | T1 | 0.367 | 0.419 | 0.538 |  |  |
|  | ≥T2 | 0.143 | 0.226 | 0.231 |  |  |
| **Lymph node metastasis** | N0 | 0.857 | 0.774 | 0.769 | 1.107 | 0.575 |
|  | ≥N1 | 0.143 | 0.226 | 0.231 |  |  |
| **Distant metastasis** | M0 | 0.857 | 0.774 | 0.769 | 1.107 | 0.575 |
|  | M1 | 0.143 | 0.226 | 0.231 |  |  |
| **Pathomorphology of nonmuscle-invasive tumors** | papillary urothelial neoplasm of low malignant potential (PUN-LMP) | 0.233 | 0.152 | 0.100 | 1.886 | 0.757 |
|  | low-grade papillary urothelial carcinoma | 0.400 | 0.394 | 0.500 |  |  |
|  | high-grade papillary urothelial carcinoma | 0.367 | 0.455 | 0.400 |  |  |

**Supplementary Table 5.** Distribution of genotypes and alleles of the c. 47 C>T (p.Val16Ala) – *SOD2* (rs4880), c.1823 C>T (p. Ser608Leu) – *NOS2* (rs2297518) and g.-1026 C>A – *NOS2* (rs2779249) and ORs with 95% CIs in non-smokers and smokers with BC.

| Genotype/Allele | NON-SMOKER (n = 104) | | | | SMOKER (n = 126) | | | |
| --- | --- | --- | --- | --- | --- | --- | --- | --- |
|  | Control (n = 68) | BC (n = 36) | Crude OR (95% CI)* | Control (n = 68) | BC (n = 36) | BC  (n = 74) | Control (n = 68) | BC (n = 36) |
|  | N (Freq.) | N (Freq.) |  |  | N (Freq.) | N (Freq.) |  |  |
| **c.47 T>C (p.Val16Ala) – *SOD2* (rs4880)** | | | | | | | | |
| C/C | 10 (0.256) | 16 (0.381) | 1.785 (0.689-4.619) | 0.233 | 19 (0.253) | 13 (0.176) | 0.628 (0.284-1.389) | 0.251 |
| T/C | 21 (0.538) | 20 (0.476) | 0.779 (0.325-1.866) | 0.576 | 35 (0.467) | 46 (0.622) | 1.878 (0.977-3.608) | 0.059 |
| T/T | 8 (0.205) | 6 (0.143) | 0.646 (0.202-2.065) | 0.461 | 21 (0.280) | 15 (0.203) | 0.654 (0.306-1.396) | 0.272 |
| χ^2^ = 80.998; *p =* 0.386 | | | | | χ^2^ = 149.000; *p =* 0.416 | | | |
| C | 41 (0.526) | 52 (0.619) | 1.493 (0.782-2.850) | 0.224 | 73 (0.487) | 72 (0.486) | 0.999 (0.621-1.608) | 0.997 |
| T | 37 (0.474) | 32 (0.381) | 0.670 (0.351-1.279) | 0.224 | 77 (0.513) | 76 (0.514) | 1.001 (0.622-1.610) | 0.997 |
| **c.1823 C>T (p. Ser608Leu) – *NOS2* (rs2297518)** | | | | | | | | |
| C/C | 27 (0.692) | 27 (0.643) | 0.800 (0.316-2.023) | 0.637 | 49 (0.653) | 55 (0.653) | 1.536 (0.758-3.111) | 0.233 |
| C/T | 11 (0.282) | 14 (0.333) | 1.273 (0.493-3.283) | 0.618 | 24 (0.320) | 18 (0.243) | 0.683 (0.333-1.402) | 0.299 |
| T/T | 1 (0.026) | 1 (0.024) | 0.927 (0.056-15.344) | 0.958 | 2 (0.027) | 1 (0.014) | 0.500 (0.044-5.636) | 0.575 |
| χ^2^ = 81.002; *p =* 0.386 | | | | | χ^2^ = 149.000; *p =* 0.416 | | | |
| C | 65 (0.833) | 68 (0.810) | 0.843 (0.368-1.930) | 0.686 | 122 (0.813) | 128 (0.865) | 1.497 (0.787-2.848) | 0.219 |
| T | 13 (0.167) | 16 (0.190) | 1.187 (0.518-2.719) | 0.686 | 28 (0.187) | 20 (0.135) | 0.668 (0.351-1.271) | 0.219 |
| **g.-1026 C>A – *NOS2* (rs2779249)** | | | | | | | | |
| C/C | 21 (0.538) | 25 (0.595) | 1.261 (0.522-3.042) | 0.606 | 36 (0.480) | 40 (0.541) | 1.275 (0.670-2.426) | 0.460 |
| C/A | 15 (0.385) | 9 (0.214) | 0.436 (0.164-1.162) | 0.097 | 36 (0.480) | 28 (0.378) | 0.659 (0.343-1.266) | 0.211 |
| A/A | 3 (0.077) | 8 (0.190) | 2.824 (0.691-11.533) | 0.148 | 3 (0.040) | 6 (0.081) | 2.118 (0.509-8.805) | 0.302 |
| χ^2^ = 80.994; *p =* 0.386 | | | | | χ^2^ = 149.000; *p =* 0.416 | | | |
| C | 57 (0.731) | 59 (0.702) | 0.896 (0.488-1.645) | 0.723 | 108 (0.720) | 108 (0.730) | 1.054 (0.621-1.790) | 0.845 |
| A | 21 (0.269) | 25 (0.298) | 1.116 (0.608-2.051) | 0.723 | 42 (0.280) | 40 (0.270) | 0.949 (0.559-1.610) | 0.845 |

* crude OR means OR calculated with conventional logistic regression; for the significant outcomes


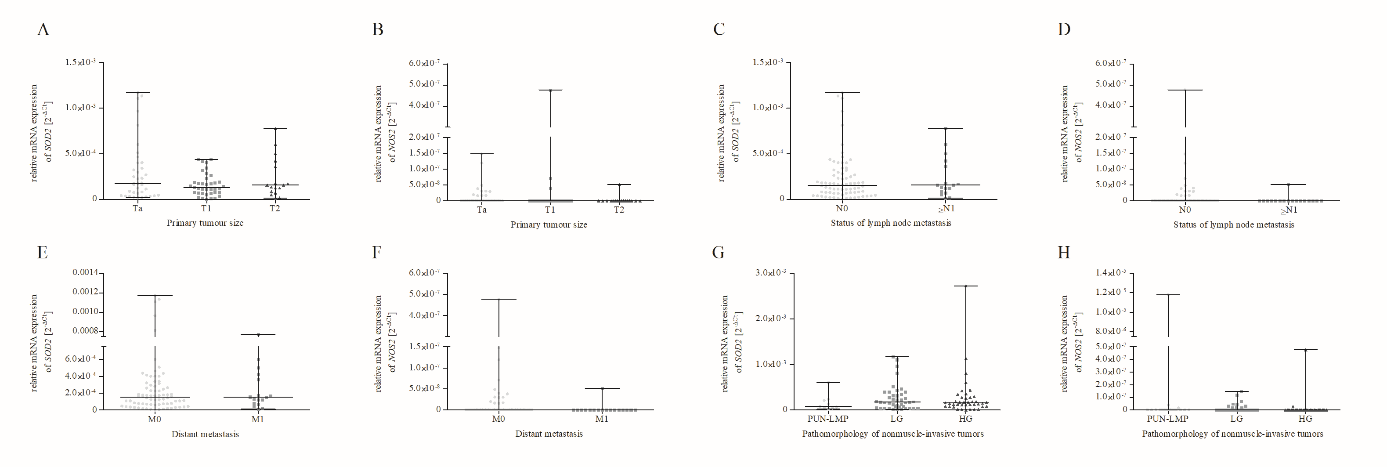


**Supplementary Figure 1.** Relative *SOD2* and *NOS2* expression in the subgroups of the primary tumour size (A, B), the lymph node metastasis (C, D), the distant metastasis (E, F), and the histopathological diagnosis (G, H). Relative mRNA are expressed as the 2^−ΔCt^ (C_t_ _gene_ – C_t_ _18S_) method for each sample. The data are plotted as individual values and the median with an interquartile range is indicated by the horizontal bars.


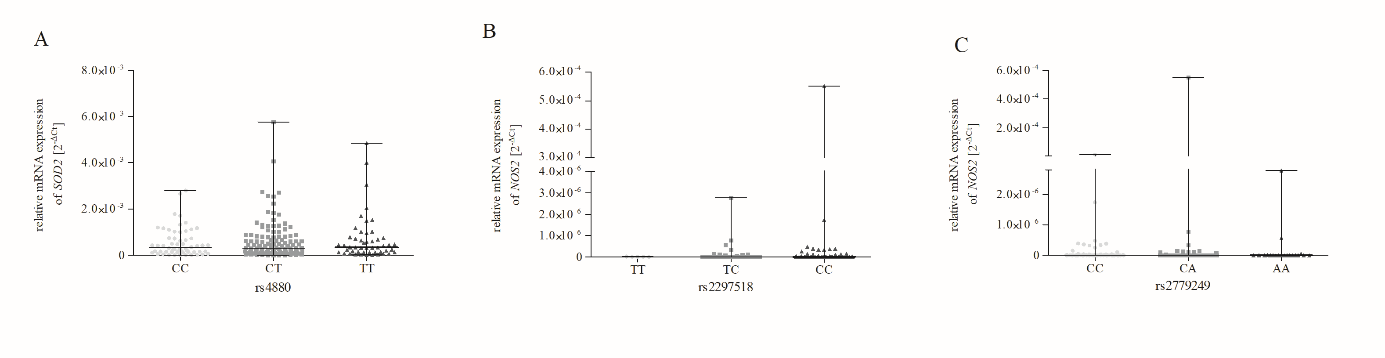


**Supplementary Figure 2.** Impact of single-nucleotide polymorphisms related to the *SOD2* gene on superoxide dismutase 2 mRNA expression (A) and *NOS2* gene on nitric oxide synthetase 2 mRNA expression (B, C) in study participants. Gene expression in PBMCs is expressed as the 2^−ΔCt^ (C_t_ _gene_ – C_t_ _18S_) method. Data are shown as scatter dot plots, horizontal lines represent the median, whereas whiskers correspond to the interquartile range.


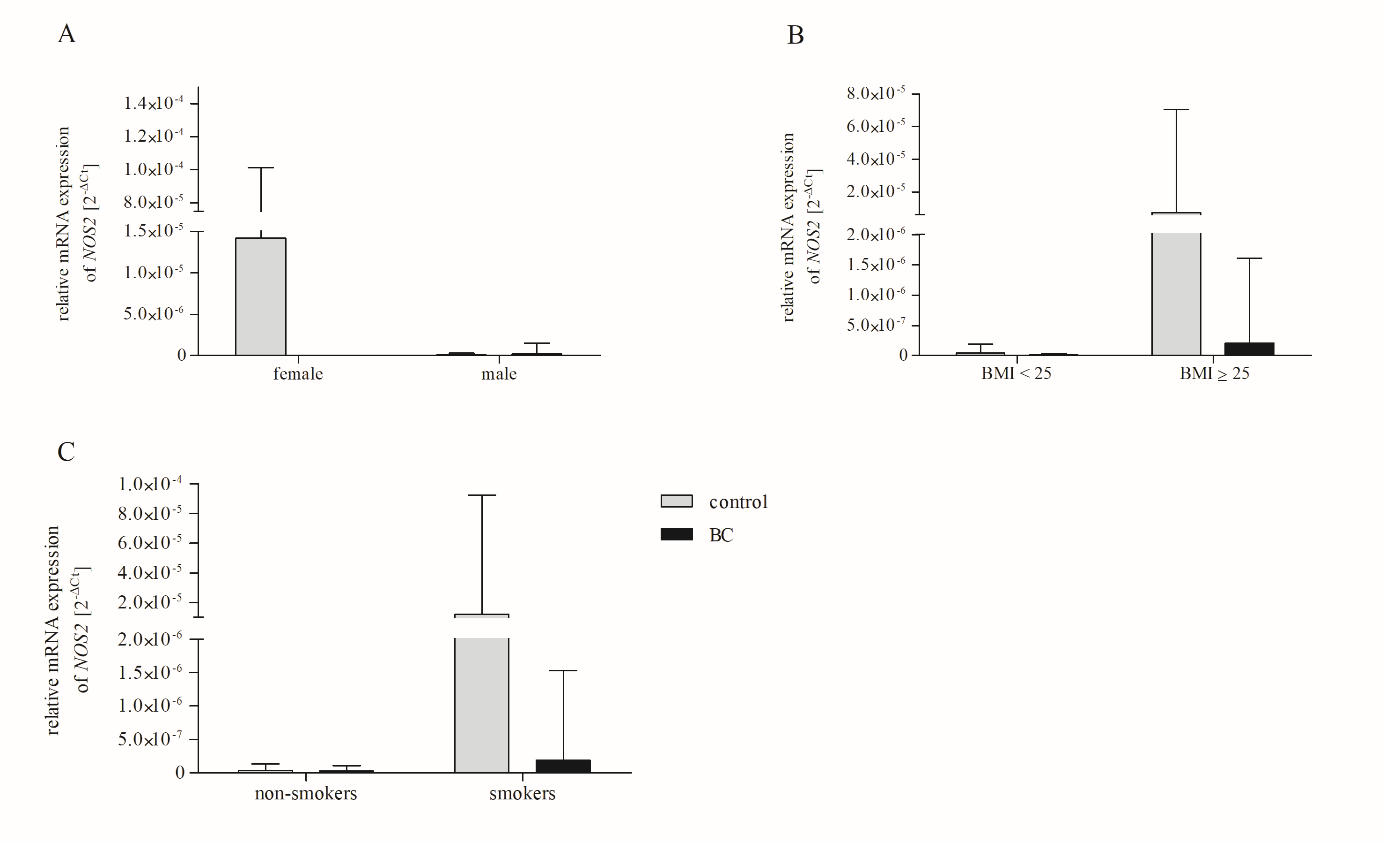


**Supplementary Figure 3.** Two-way ANOVA with Bonferroni post hoc test shows signiﬁcant effects of gender (A), BMI (B), cigarette smoking (C), and BC on the mRNA expression of *NOS2*. Gene expression in PBMCs is expressed as the 2^−ΔCt^ (C_t_ _gene_ – C_t_ _18S_) method. The data are presented as mean ± SD.

**Supplementary Figure 4.** Methylation status of *SOD2* and *NOS2* promoter regions in the subgroups of the primary tumour size (A, B), the lymph node metastasis (C, D), the distant metastasis (E, F), and the histopathological diagnosis (G, H). The data are plotted as individual values and the median with an interquartile range is indicated by the horizontal bars.
